# Regulation of brain endothelial cell physiology by the TAM receptor tyrosine kinase Mer

**DOI:** 10.1038/s42003-023-05287-y

**Published:** 2023-09-07

**Authors:** Kaisa E. Happonen, Patrick G. Burrola, Greg Lemke

**Affiliations:** Molecular Neurobiology Laboratory, The Salk Institute for Biological Studies, La Jolla, CA 92037 USA

**Keywords:** Blood-brain barrier, Neuro-vascular interactions

## Abstract

The receptor tyrosine kinase Mer (gene name *Mertk*) acts in vascular endothelial cells (ECs) to tighten the blood-brain barrier (BBB) subsequent to viral infection, but how this is achieved is poorly understood. We find that Mer controls the expression and activity of a large cohort of BBB regulators, along with endothelial nitric oxide synthase. It also controls, via an Akt-Foxo1 pathway, the expression of multiple angiogenic genes. Correspondingly, EC-specific *Mertk* gene inactivation resulted in perturbed vascular sprouting and a compromised BBB after induced photothrombotic stroke. Unexpectedly, stroke lesions in the brain were also reduced in the absence of EC Mer, which was linked to reduced plasma expression of fibrinogen, prothrombin, and other effectors of blood coagulation. Together, these results demonstrate that Mer is a central regulator of angiogenesis, BBB integrity, and blood coagulation in the mature vasculature. They may also account for disease severity following infection with the coronavirus SARS-CoV-2.

## Abstract (web summary)

Transcriptomics and proteomics studies show that the endothelial cell-specific genetic inactivation of Mer leads to pleiotropic deficits in angiogenesis, blood brain barrier formation, and blood coagulation.

## Introduction

Macrophages and other sentinel cells of the immune system express the TAM receptor tyrosine kinases (RTKs) Mer and Axl^1–4^. In these cells, TAM signaling, activated by binding of the ligands Gas6 and Protein S (Pros1)^5^, mediates the phagocytosis of phosphatidylserine-displaying apoptotic cells (ACs) and membranes^2,4,6^, and simultaneously, acts as a cell-intrinsic feedback inhibitor to suppress Toll-like and cytokine receptor signaling at the termination of the innate immune response^1,3,7^. These activities have recently been the subject of intense study in microglia, the specialized tissue macrophages of the central nervous system (CNS), in the context of both normal brain homeostasis and neurodegenerative disease^8–11^.

This microglial focus notwithstanding, TAM signaling exerts potent regulatory activities in a second population of cells in the CNS—the endothelial cells (ECs) of the brain vasculature. We have previously shown that brain ECs prominently express Mer^8,12^, and that this RTK is required for tightening the BBB in the face of systemic flavivirus and orthobunyavirus infections^12^. *Mertk*^*−/−*^ mouse mutants^13,14^, or wild-type (WT) mice treated with a TAM tyrosine kinase inhibitor (BMS 777607)^15^, are hypersusceptible to lethal infection of the CNS by West Nile virus and La Crosse virus, both of which enter the brain from the blood^12^. We demonstrated that this enhanced vulnerability occurs because Mer normally acts to tighten the BBB by signaling in concert with the type I interferon receptor in ECs. We found that these signaling systems together enhance transendothelial electrical resistance and tight junction integrity via their ability to drive remodeling of the actin cytoskeleton at EC tight junctions^12^.

Although these results demonstrated that Mer regulates EC function in the cerebral vasculature in vivo, the signal transduction pathways downstream of Mer activation through which this regulation is achieved are not well understood. We therefore interrogated these pathways in mouse and human brain vascular ECs. Using a combination of phospho- and total proteomic mass spectrometry, RNA sequencing, germline and EC-specific *Mertk* gene ablation, and pharmacological inhibition of endothelial Mer tyrosine kinase activity in vitro and in vivo, we found that this RTK regulates batteries of genes and proteins that control three essential functions in adult ECs. The first of these is the organization of the actin cytoskeleton at tight junctions. The second is the regulation of genes that control vascular development and angiogenesis. The third is plasma expression of a set of proteins that regulate fibrin metabolism during blood coagulation. Correspondingly, loss of Mer in the endothelium had major consequences in the context of experimentally induced stroke. Although we have focused our studies on the vascular ECs of the brain, Mer is expressed by all mouse and human ECs^16,17^. It is therefore likely that the findings we describe extend to all of the blood vessels of the body.

## Results

### Mer regulation of protein phosphorylation

As demonstrated previously^8^ and is highlighted in Supplementary Fig. 1a, mouse brain vascular ECs (BECs) and microglia are prominent sites of Mer expression in the mature CNS. Many of our analyses were performed with BECs isolated from young adult (8–12 week) mice, and maintained in vitro as described previously^12,18^ (see Methods). These cells express abundant Mer protein and *Mertk* mRNA (Supplementary Fig. 1a–d)^12,17^, very little Axl protein but detectable *Axl* mRNA, no protein or mRNA for Tyro3, the other TAM receptor, and appreciable mRNA for both Mer ligands—Gas6 and Pros1 (Supplementary Fig. 1b–d)^17^. The fact that neither WT nor *Mertk*^*−/−*^ mouse BECs express Tyro3 is important, since it has been shown that: (a) Tyro3, whose gene is very closely linked to the *Mertk* gene in the mouse genome, cooperates with Mer to regulate phagocytosis in retinal pigment epithelial (RPE) cells, where both of these TAM receptors are expressed^19–21^; and (b) the *Mertk*^*−/−*^ mutation we have employed markedly lowers Tyro3 expression in these cells^20,21^. Although this mutation also perturbs RPE expression of several other linked genes (e.g., the *Gchfr*, *Exd1*, *Duoxa2*, and *Aa467197* genes)^21^, only *Tyro3* has been shown to influence *Mertk* function in the RPE^19,21^. As described below, we also carried out experiments with human BECs (see Methods), which express both Mer and Axl (Supplementary Fig. 1e). Although other ECs in culture, including mouse and porcine aortic ECs and human umbilical vein ECs (HUVECs), also express Axl^22–24^ and *Axl*^*−/−*^ mutants have a leaky vasculature in the liver^25^, our prior analyses with *Mertk*^*−/−*^ and *Axl*^*−/−*^ mutants^12^ indicated that Mer is the only physiologically significant TAM receptor with respect to barrier function in the mouse brain. Moreover, single cell mouse transcriptomic data (https://endotheliomics.shinyapps.io/ec_atlas/) demonstrate that *Axl* mRNA is expressed in only select EC populations in the body (the liver most prominently, the brain not at all), whereas *Mertk* mRNA is expressed by all ECs. Finally, in studies with human BECs described below, we found that key regulatory phenomena were controlled by Mer and not by Axl. We therefore focused our analyses on Mer.

**Fig. 1:**
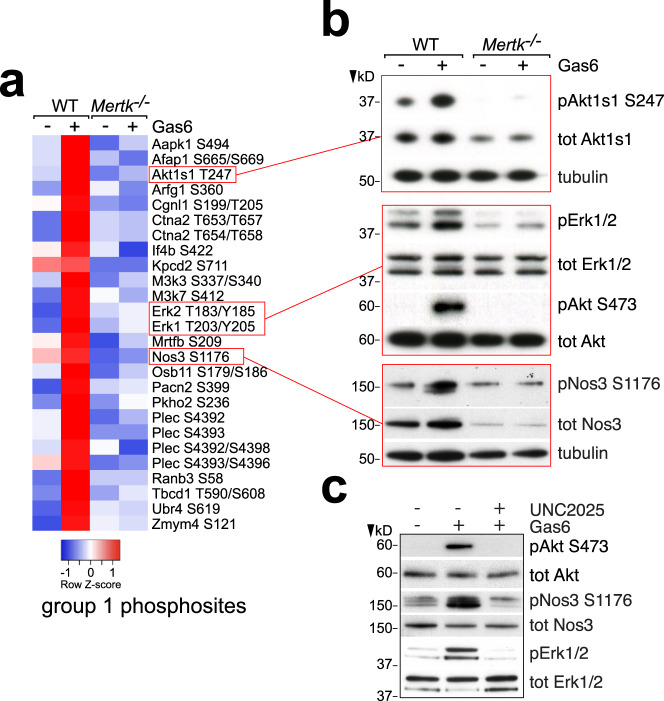
Mer-dependent phosphorylation in ECs. **a** Phosphoproteomic heat map of selected group 1 phosphosites whose phosphorylation was strongly induced in mouse BECs by 15 min incubation with 10 nM Gas6, and not in *Mertk*^*−/−*^ BECs (see Methods). **b** Representative western blot confirmation of selected results in (**a**) using the indicated phospho-specific antibodies. **c** Representative western blot demonstration that UNC2025 (300 nM, added 30 min prior Gas6) inhibits Gas6-induced phosphorylation (10 nM, 15 min) of Akt, Nos3, and Erk1/2 in WT mouse BECs. (Quantification of multiple runs for these and other western blots in the paper are presented in Supplementary Fig. 9).

The Mer tyrosine kinase is activated by the binding of either Gas6 or Pros1 to the receptor ectodomain^5^. [Of relevance to ECs, Pros1 is present at high concentrations (~300 nM) in the blood, whereas Gas6 is not; and Axl can only be stimulated by Gas6^5^]. Although Mer activation has been shown to result in the engagement of Src family kinases and the subsequent activation of downstream kinases in HEK-293T cells, platelets, and cancer cell lines, a systematic analysis of Mer signaling has not been performed in any cell type. We therefore used phosphoproteomic mass spectrometry (phospho-MS) to carry out a preliminary assessment of both rapid and long term phosphorylation events triggered by Mer activation in mouse BECs. We measured phosphorylation sites (serine-threonine primarily) in lysates prepared from WT or *Mertk*^*−/−*^ BECs^13^ that were treated or untreated with mouse Gas6 (10 nM) for 15 min (min; see Methods). We detected 186 phosphopeptides showing differential abundance between samples, distributed over 133 different proteins. We segregated these into those whose phosphorylation was: (a) induced by Gas6 in WT but not in *Mertk*^*−/−*^ BECs (group 1, Fig. 1a); (b) repressed by Gas6 in WT but not in *Mertk*^*−/−*^ BECs (group 2, Supplementary Fig. 2b); (c) not regulated by Gas6 in WT or *Mertk*^*−/−*^ BECs but reduced in *Mertk*^*−/−*^ cells (group 3, Supplementary Fig. 2c); and (d) not regulated by Gas6 in WT or *Mertk*^*−/−*^ BECs but elevated in *Mertk*^*−/−*^ BECs (group 4, Supplementary Fig. 2d). (Source data for all analyses in this paper can be found in Supplementary Data 1).

Most of the group 1 sites (73%) were located in proteins that fell into just two categories: components of kinase signaling cascades, and components or regulators of the cytoskeleton. In the former, the T183/Y185 sites of MAP kinase 1 (Erk2) and T203/Y205 sites of MAP kinase 3 (Erk1), the S494 site of AMP kinase (Aapk1), the T247 site of Akt substrate 1 (Akt1s1), and the S337/S340 site of MAPK kinase kinase 3 and S412 site of MAPK kinase kinase 7 are all well-described phosphorylation sites downstream of the activation of many RTKs (Fig. 1a). Mer-dependent phosphorylation of the Akt1s1 and Erk1/2 sites was confirmed by western blotting (Fig. 1b). Although phospho-MS did not detect phosphorylation of the key downstream kinase Akt, western blots revealed Gas6-stimulated phosphorylation of the canonical Akt activation sites T308 and S473^26^ (Fig. 1b). As discussed below, many of the events downstream of Mer activation are dependent on its ability to phosphorylate Akt.

Among the group 1 phosphosites detected in EC cytoskeletal proteins (Fig. 1a) were: the S665/S669 sites of the actin filament associated protein 1 (Afap1); the S199/T205 sites of paracingulin (Cgnl1); the T653/T657 and T654/T658 sites of catenin alpha 2; and the S4392, S4393, S4392/S4398, and S4393/4396 sites of plectin. The phosphorylation of these cytoskeletal proteins is consistent with (a) the observed ability of activated Mer to reorganize tight junction components in vascular ECs in response to virus^12^, (b) the reconfiguration of the actin cytoskeleton driven by Mer and Axl during macrophage phagocytosis of ACs^2,4^, and (c) the phenomena described below. A final group 1 phosphosite—S1176—was detected in endothelial nitric oxide synthase (eNOS, Nos3), which is among the most powerful regulators of EC physiology^27^. Phosphorylation at S1176, which in ECs is catalyzed by Akt, activates eNOS^27^. We used the Akt inhibitor Miransertib to confirm that Gas6-induced eNOS phosphorylation in BECs was mediated by Akt (Supplementary Fig. 2a).

For many of our analyses, we have additionally assessed the effects of acute pharmacological inhibition of Mer tyrosine kinase activity in comparison to those seen with genetic *Mertk* ablation, using UNC2025, a water-soluble small-molecule inhibitor that antagonizes the tyrosine kinase activity of both Mer and Flt3 with an IC_50_ of <1 nM and that shows >20-fold selectivity over Axl and Tyro3^28,29^. [Mouse BECs do not express Flt3 (Supplementary Fig. 1f).] This inhibition of Mer enzymatic activity in WT cells is particularly important as an independent experimental adjunct and confirmation of genetic analyses, since any engineered perturbation to the genome may affect the expression of downstream or closely-linked genes in addition to the one targeted. As noted above, the original germline *Mertk*^*−/−*^ mutation has been shown to markedly lower expression of the closely-linked *Tyro3* gene in mouse RPE cells^19–21^, and to alter the expression (albeit more modestly) of several additional closely-linked genes in these cells^21^. As shown in Fig. 1c, 30 min pre-incubation with UNC2025 completely blocked Gas6 stimulation of the phosphorylation of Akt, eNos, and Erk1/2 in WT BECs.

Only four group 2 phosphosites—those whose expression was repressed by Gas6 in WT BECs but not in *Mertk*^*−/−*^ cells—were identified (Supplementary Fig. 2b). Consistent with their Gas6-mediated down-regulation, phosphorylation of S192 in Csrp1 and of S352 in Catenin delta 1 (Ctdn1, p120-catenin) was detected. Phospho-MS detected 160 EC phosphosites that were not regulated within 15 min of Gas6 addition, but that were nonetheless Mer-dependent (Supplementary Fig. 2c, d). There were 42 sites in 20 proteins in group 3 (reduced in *Mertk*^*−/−*^ BECs), and 118 sites in 71 proteins in group 4 (elevated in *Mertk*^*−/−*^ BECs). A large fraction of the group 3/4 phosphosites were again present on cytoskeleton-associated proteins (Supplementary Fig. 2e). Among many others, these included: the T1636 and S1634/S1648 sites of the microtubule-associated protein Map1a, and the S1391/S1395 and S1497 sites of Map1b; the S55, S56, and S459 sites of vimentin; the T513/T512, T513/S516, and S618 sites of the dynein light intermediate chain (Dc1li1); the S1734, T1747, T1750, S2152, and S2327 sites of filamin A, and the S518, S519, and S789 sites of filamin B; the Y118, S250, and S258/S261 sites of the focal adhesion protein paxillin; and the S729, S979, S1328, and S2040 sites of talin. Again, the ability of Mer to drive changes in the phosphorylation of so many cytoskeletal proteins, is consistent with the observed ability of this RTK to drive the rapid changes in actin cytoskeletal organization seen during tightening of the BBB by vascular ECs^12^, and with the rapid time course of Mer-driven formation and actuation of the phagocytic cup during the engulfment of apoptotic cells by macrophages^2^.

### Mer regulation of EC gene and protein expression

We next assessed the effects of genetic versus pharmacological Mer inactivation with respect to mouse BEC mRNA and protein. In this regard, it is important to emphasize that neither Mer nor Axl play any observable role in vascular development in the embryo. These RTKs are only minimally expressed in the embryo, are markedly up-regulated postnatally throughout the body, and are then maintained at high levels in the adult^30,31^. Accordingly, vessel density, length, branching, and lacunarity in adult WT versus *Mertk*^*−/−*^ brains were all unchanged between genotypes (Supplementary Fig. 3a–e). This stands in contrast to other RTKs and RTK ligands that regulate EC physiology, including the vascular endothelial growth factors (VEGFs), the VEGF receptors, and the Tie1/2 receptors. These proteins play essential roles in vasculogenesis—the formation of blood vessels during embryogenesis - and mice carrying loss-of-function mutations in their genes are often embryonic lethals, in some instances lacking a vasculature entirely^32,33^.

**Fig. 2:**
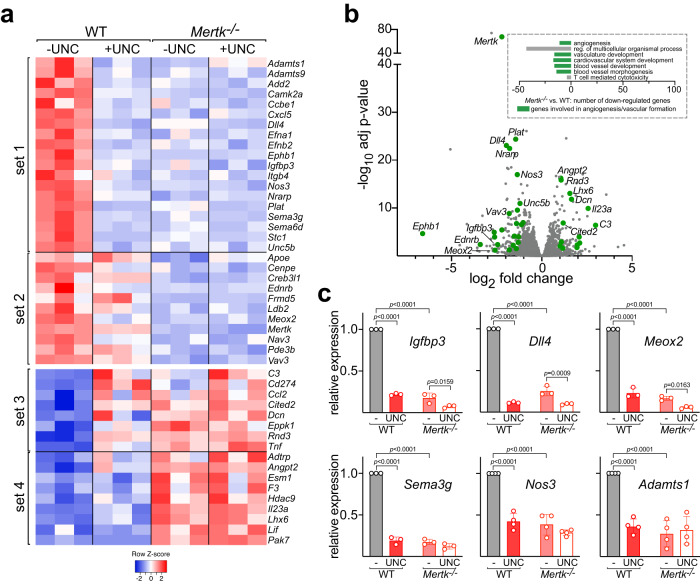
Mer-dependent regulation of angiogenic genes in mouse BECs. **a** Heat maps of results from RNA-seq analysis of WT versus *Mertk*^*−/−*^ BECs, without or with 20 h pretreatment with 300 nM UNC2025, curated for angiogenic genes. Set 1, genes whose expression was reduced in both UNC-treated and *Mertk*^*−/−*^ BECs; set 2, genes not reduced by UNC but reduced in *Mertk*^*−/−*^ BECs; set 3, genes whose expression was induced in both UNC-treated and *Mertk*^*−/−*^ BECs; set 4, genes not induced by UNC but induced in *Mertk*^*−/−*^ BECs. **b** Volcano plot of up (0–5, *x*-axis) and down (0 to −5)-regulated mRNAs isolated from *Mertk*^*−/−*^ versus WT BECs, with angiogenic genes highlighted in green. **c** Confirmation of RNA-seq results by qRT-PCR (see Methods) for selected mRNAs. Results displayed are mean ±1 standard deviation for the indicated number of samples analyzed; and *p* values are indicated only for statistically significant differences. Statistical significance in (**c**) was evaluated using a two-way ANOVA with a Tukey’s multiple comparisons test.

We cultured WT or *Mertk*^*−/−*^ mouse BECs in the absence or presence of the Mer inhibitor UNC2025 for 20 h prior to isolating RNA for bulk RNA-sequencing (see Methods). We thereby identified four distinct gene sets (Fig. 2a), those whose expression was: (a) down-regulated both by Mer kinase inhibition and by *Mertk* gene mutation (set 1); (b) not affected by Mer kinase inhibition but down-regulated in *Mertk*^*−/−*^ BECs (set 2); (c) up-regulated both by Mer inhibition and by *Mertk* gene mutation (set 3); and minimally or not affected by Mer inhibition but up-regulated in *Mertk*^*−/−*^ BECs (set 4). A gene ontology (GO) pathway analysis demonstrated that five out of seven pathways among genes down-regulated in *Mertk*^*−/−*^ versus WT BECs were related to angiogenesis, and 30 of the 172 downregulated genes matched the GO-terms angiogenesis and/or migration (Fig. 2b, inset). We therefore focused our attention on this subset, heat maps of representative members of which are illustrated in Fig. 2a. A volcano plot of up- and down-regulated mRNAs, with genes of specific relevance to EC angiogenesis highlighted in green, is presented in Fig. 2b. The relative expression level for all identified angiogenic genes is displayed in Supplementary Fig. 4.

These analyses revealed that Mer signaling controls the expression of a large suite of genes whose products coordinate adult angiogenesis – the development of new blood vessels (Fig. 2a). Examples of set 1 mRNAs include those encoding: the zinc-dependent metalloproteinases Adamts1 and 9, which modulate angiogenesis in multiple settings; Ccbe1, which is required for the proteolytic activation of VEGF-C by Adamts3; the Notch ligand Delta-like 4 (Dll4), which (as discussed below) is essential for the specification of endothelial “tip cells” and sprouting angiogenesis^34,35^; the Eph RTK EphB1 and the Eph ligands ephrin-A1 and B2, which are induced downstream of Dll4-Notch activation; the insulin-like growth factor binding protein 3 (Igfbp3); nitric oxide synthase 3 (Nos3, eNOS) discussed above; the Notch-regulated ankyrin repeat protein Nrarp; the Plexin ligands semaphorin 3G and 6D (sema3g/6d); and the vascular Netrin receptor Unc5b, whose expression is upregulated during sprouting angiogenesis^36^. Both Mer tyrosine kinase activity and Mer protein were required for BEC expression of all of these genes (Fig. 2a). This regulation was confirmed for several mRNAs using qRT-PCR (Fig. 2c). Two set 2 genes exhibited modestly reduced expression at 20 h after treatment with UNC2025 (Fig. 2a). Although both of these genes—Ednrb (the endothelin receptor type B) and Meox2 (a homeodomain transcription factor)—encode proteins with regulatory functions in ECs, their expression level in mouse BECs was exceptionally low (Supplementary Fig. 4a). As expected, *Mertk* was a set 2 gene^2,13^.

Several set 3 genes—those whose expression was elevated by both UNC2025 and *Mertk* mutation—encode known inhibitors of angiogenesis. Included in this group is Cited2, which strongly inhibits transcription of VEGF-A and other neoangiogenic genes^37^. One of the most prominently regulated genes in set 4, those upregulated by *Mertk* deletion but not altered by short-term Mer inhibition, was angiopoietin-2 (Angpt2), a naturally occurring antagonist of the binding of proangiogenic angiopoietin-1 to its receptor Tie2^38^. Finally, we examined a set of ten genes that are closely-linked to *Mertk* in the mouse genome, since, as noted above, expression of several of these genes has been shown to be perturbed by the *Mertk* mutation^21^. Only one of these genes— *Aa467197*, encoding a protein of unknown function - was expressed by mouse BECs (Supplementary Fig. 4c). *Aa467197* expression was reduced ~4-fold in *Mertk*^*−/−*^ BECs, but an equivalent reduction was seen in UNC2025-treated WT BECs (Supplementary Fig. 4c), rendering *Aa467197* a set 1 gene. In sum, pharmacological and/or genetic inhibition of Mer signaling resulted in the reduced expression of many proangiogenic genes, and concomitantly, in the enhanced expression of several antiangiogenic genes.

We extended these transcriptomic analyses by carrying out total proteome MS studies in WT versus *Mertk*^*−/−*^ mouse BECs (Supplementary Fig. 5). Of the 3787 proteins detected (far fewer proteins were detected by MS than mRNAs by RNA-seq), 163 were downregulated in *Mertk*^*−/−*^ BECs compared to WT, and 39 of these were associated with regulation of angiogenesis or migration, including Nos3 (eNOS) and Ats1 (Adamts1) (Supplementary Fig. 5a). Panther GO pathway enrichment analysis of downregulated proteins further showed an overrepresentation of proteins related to intermediate filament organization (Supplementary Fig. 5b). This is consistent with the Mer-dependent regulation of the phosphorylation of many cytoskeletal proteins documented above (Fig. 1, Supplementary Fig. 2). Remarkably, we found that nearly all structural components of the vascular basement membrane—including perlecan (Hspg2), nidogen 1 and 2, Sparc, and laminins a4, a5, b1, b3, and c1— were significantly diminished in *Mertk*^*−/−*^ BECs relative to WT (Supplementary Fig. 5a). Since basement membrane integrity is crucial for BBB maintenance, this dysregulation may contribute to the weakened BBB in *Mertk*^*−/−*^ mice^12^. Finally, these MS analyses highlighted Mer regulation of BEC expression of several proteins that act during blood coagulation (Supplementary Fig. 5a). (MS was unable to detect expression of tissue plasminogen activator under any condition, although *Plat1* mRNA was markedly downregulated in both *Mertk*^*−/−*^ and UNC2025-treated BECs (Fig. 2a).) Mer regulation of coagulation proteins may be especially relevant to the in vivo phenomena described below.

### Mer regulation of Foxo1 translocation via Akt

**Fig. 3:**
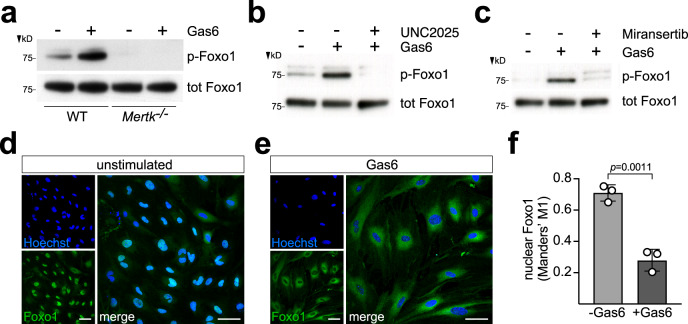
Mer and Akt dependence of Foxo1 phosphorylation and cytoplasmic versus nuclear localization in BECs. **a** Representative western blot of Foxo1 phosphorylated on S256 (p-Foxo1) and total Foxo1 in extracts of WT and *Mertk*^*−/−*^ BECs untreated (−) or treated (+) for 15 min with 10 nM Gas6. **b** Representative western blot, probed as in (**a**), of extracts of WT BECs stimulated (15 min) with 10 nM Gas6, untreated (−) or treated (+) with the Mer kinase inhibitor UNC2025 (300 nM, 30 min prior to Gas6). **c** Representative western blot, probed as in (**a**), of extracts of WT BECs stimulated (15 min) with 10 nM Gas6, untreated (−) or treated (+) with the Akt kinase inhibitor Miransertib (100 nM, 30 min prior to Gas6). Representative blots from BEC cultures from three independent isolations. **d**, **e** Images of representative field of BECs ∓ 10 nM Gas6 (30 min), and stained for nuclei (Hoechst, blue) and Foxo1 (green). Right-most images are 2× merged panels. All scale bars are 50 μm. **f** Quantification of Foxo1 nuclear localization ∓ Gas6. (Manders’ M1 % is the percentage of green pixels that overlap with blue in these images.) The graph shows the mean ±1 SD of BEC cultures from three independent isolations; >1200 cells imaged per condition. Statistical significance in (**f**) was evaluated using an unpaired *t* test. (Quantification of multiple western blot runs in Supplementary Fig. 9).

Mer’s ability to simultaneously induce proangiogenic genes and repress antiangiogenic genes suggests that it controls the expression or activity of one or more bifunctional transcription factors. In ECs, among the most important of such proteins are members of the Foxo family, most notably Foxo1^39^. We found that although Foxo1 expression was unaffected by *Mertk*^*−/−*^ mutation, both basal and Gas6-stimulated Foxo1 phosphorylation on S256, a site that controls Foxo1 subcellular localization, were eliminated in *Mertk*^*−/−*^ BECs (Fig. 3a). This same dependence was seen when WT BECs were treated with UNC2025 for 30 min prior to Gas6 application (Fig. 3b). We further found that the principal Mer-activated kinase that phosphorylates Foxo1 in BECs was again Akt, since 30 min preincubation with Miransertib substantially reduced Gas6-stimulated phosphorylation (Fig. 3c). This is consistent with prior demonstrations that Akt is the principal kinase that phosphorylates Foxo1 in most cells^40^.

Phosphorylation controls Foxo1 activity by regulating its subcellular localization: non-phosphorylated Foxo1 is nuclear, whereas phosphorylated Foxo1 is cytoplasmic^41^. We found that in unstimulated BECs, most Foxo1 was nuclear (Fig. 3d), but after 30 min incubation with Gas6, most Foxo1 was cytoplasmic (Fig. 3e). Thus, activated Mer eliminates Foxo1-regulated transcription by driving Foxo1 from the BEC nucleus. Results similar to these have previously been seen in HUVECs subsequent to Gas6 activation of the TAM receptor Axl^42^. Given that Foxo1 represses transcription of many proangiogenic genes and activates transcription of many antiangiogenic genes, the ability of Mer to drive Foxo1 from the nucleus accounts for the differential effects of *Mertk* mutation on genes that positively versus negatively regulate angiogenesis. For example, expression of the proangiogenic *Nos3* was markedly reduced both by UNC2025 treatment of WT BECs and in *Mertk*^*−/−*^ BECs, and correspondingly, the *Nos3* gene is repressed by Foxo1 in ECs^43^. Similarly, BEC expression of the proangiogenic *Dll4* and *Adamts1* genes is also repressed by Foxo1, which is relieved via Akt-mediated Foxo phosphorylation^44^. Conversely, the partial Tie2 agonist angiopoietin-2 (Angpt2) is in many settings antiangiogenic, and *Angpt2* mRNA was the most abundant (Supplementary Fig. 2b) of the mRNAs that were induced in *Mertk*^*−/−*^ BECs (Fig. 2a, b). Correspondingly, the *Angpt2* gene is normally activated by Foxo1 in ECs^43^. Similarly, the antiangiogenic gene *Cited2* is also activated by Foxo1 in ECs^45^, and its expression was elevated by *Mertk* mutation and by UNC2025 treatment. Mer regulation of all of these mRNAs again appeared to be mediated by Akt, as it was inhibited by Miransertib (examples in Supplementary Fig. 2f). A model of the differential effects of Mer-mediated Foxo1 phosphorylation on the expression of pro- versus antiangiogenic genes is illustrated in Supplementary Fig. 2g.

### Mer interaction with VEGF signaling

The most intensively investigated angiogenic proteins are the five VEGFs, of which VEGF-A is of paramount importance to vessel formation^46^. There have been earlier, conflicting studies of the interaction of VEGF-A and TAM receptor signaling in cultured ECs. Ruan and Kazlauskas found that inactivation of Axl in HUVECs, porcine aortic ECs, or mouse heart ECs resulted in diminished activation of Akt by VEGF-A^22^. In contrast, Gallicchio and colleagues found that Gas6 binding to Axl inhibited both VEGF-A-stimulated activation of VEGF receptor 2 and VEGF-A-stimulated chemotaxis in HUVECs^47^. And Fraineau et al. found that Pros1 modestly inhibited VEGF-A-mediated Erk1/2 phosphorylation, Akt phosphorylation, and cell migration, also in HUVECs^48^.

We first asked whether the Gas6 effects described above required VEGFR activation. We antagonized VEGFR signaling with Axitinib, a potent small-molecule inhibitor of the tyrosine kinases of all VEGF receptors (and of the PDGF receptor and c- kit^49^). While 30 min pre-incubation with Axitinib inhibited VEGF-A-driven phosphorylation of both eNOS and Erk1/2 in mouse BECs as expected (Supplementary Fig. 6a), it had no effect on Gas6-driven phosphorylation of eNOS, Erk1/2, Akt, or Foxo1 (Supplementary Fig. 6b). Thus, TAM signaling in mouse BECs does not require an active VEGF receptor. Conversely, we found that the ability of VEGF-A to stimulate rapid eNOS phosphorylation was preserved in *Mertk*^*−/−*^ BECs, even though total eNOS levels were, as shown above, markedly reduced (Supplementary Fig. 6c). Similarly, the ability of VEGF-A to stimulate Erk1/2 phosphorylation was also maintained in *Mertk*^*−/−*^ BECs (Supplementary Fig. 6d). Together, these data indicate that the signaling pathways activated by Gas6 and VEGF, while similar in configuration, operate independently in mouse BECs. In addition, as noted above, the VEGF receptors are expressed and function in ECs during vascular development in the embryo, whereas Mer is not and does not.

### Mer regulation of EC migration and morphogenesis

**Fig. 4:**
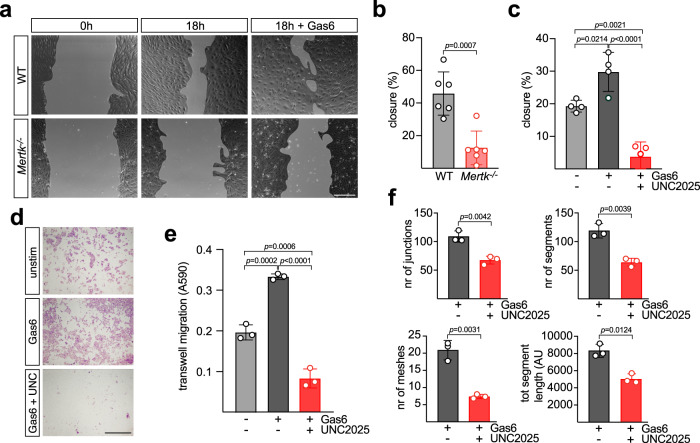
Mer regulation of BEC physiology in vitro. **a** Representative images of scratch assays performed in WT (top panels) or *Mertk*^*−/−*^ (lower panels) BECs over 18 h in the absence or presence of 10 nM Gas6. **b** Quantification of scratch closure (see Methods) in WT versus *Mertk*^*−/−*^ BECs in six independent experiments. **c** Quantification of scratch closure in WT BECs ∓ Gas6 (10 nM) and ∓UNC2025 (300 nM) in the indicated number of experiments. **d** Representative Crystal Violet staining of migrated BECs on the upper surface of a transwell membrane after 20 h unstimulated (top), with 10 nM Gas6 (middle), or with 10 nM Gas6 and 300 nM UNC2025 (bottom). **e** Quantification of transwell migration results as in (**e**) (see Methods), for three independent experiments. **f** Quantification—performed using Fiji software with the Angiogenesis Analyzer plugin - of the number of endothelial junctions (top left), segments (top right), meshes (lower left), and total segment length (lower right) 5 h after BECs were added to Geltrex basement membrane in the presence of 10 nM Gas6, ∓300 nM UNC2025. In all panels, data show the mean ±1 SD, and data points indicate independent experiments performed on separate batches of isolated BECs. Statistical significance of differences was evaluated with an unpaired t test (**b**, **f**) and with a one- way ANOVA using a Dunnett’s post test (**c**, **e**). Scale bars: **a** 200 μm and **d** 1 mm.

Given the above results, we examined the effects of Mer inactivation with respect to cultured BEC physiology. In migration assays in which a scratch was introduced across a confluent BEC monolayer (see Methods), we found that WT cells migrated and substantially sealed this scratch over 18 h, but that scratch closure was severely blunted in *Mertk*^*−/−*^ cells (Fig. 4a, b). Moreover, although Gas6 and Protein S (Pros1) are both expressed by BECs and most other ECs^17^, WT BEC migration and scratch closure was stimulated by the addition of 10 nM Gas6 (Fig. 4a, c). Both basal and Gas6-stimulated migration of WT BECs were blocked by incubation with UNC2025 at 300 nM, introduced 30 min prior to the induction of the scratch and maintained during the assay (Fig. 4c).

We also performed transwell assays in which WT BECs were seeded into the top chamber of a cell culture insert, allowed to migrate across the membrane for 18 h, and then scored for extent of migration to the opposite side of the membrane using a colorimetric assay (see Methods). Migration could again be stimulated by Gas6 added to the lower well of the chamber, and this migration was blocked by UNC2025 (Fig. 4d, e). Finally, BECs were seeded on top of Geltrex basement membrane matrix in the presence of 10 nM Gas6 and then scored for the formation of endothelial tube segments, junctions, meshes, and nodes after 5 h. All of these measures of EC morphogenesis in vitro were also significantly diminished by incubation with UNC2025 (Fig. 4f). Thus, in all of these assays, loss of the TAM receptor Mer, or pharmacological inhibition of its tyrosine kinase activity, resulted in reduced EC motility and morphogenetic capacity in vitro.

### Mer regulation in human BECs

TAM functions in human ECs have been studied in the context of HUVECs^16,48^ and aortic ECs^16^ among others, but have been focused almost entirely on Axl. In contrast to mouse BECs, human BECs (huBECs) express both Mer and Axl (Supplementary Fig. 1e). As we saw for mouse BECs, Gas6 stimulation (10 nM, 15 min) triggered phosphorylation of ERK1/2, AKT, eNOS, and FOXO1 in huBECs, and these phosphorylations were Mer-dependent, as they were abolished by UNC2025 (Supplementary Fig. 7a). A 30 min pre-incubation with Miransertib inhibited both Gas6-mediated FOXO1 and eNOS phosphorylation, and so these targets are also downstream of AKT in huBECs (Supplementary Fig. 7b). Treating huBECs for 20 h with either UNC2025 or Miransertib was further found to inhibit the expression of many Mer-regulated angiogenic genes, as seen in mouse BECs, including *NOS3*, *DLL4*, and *ADAMTS1*, while upregulating expression of the antiangiogenic *CITED2* (Supplementary Fig. 7c).

We also performed scratch assays, pre-treating huBECs with UNC2025 for either 30 min or 24 h before scratch induction, and measuring the sealing of the scratch 5 h later. UNC2025 inhibited huBEC migration when administered 24 h prior to scratch induction, whereas a 30 min pre-incubation produced only marginal inhibition (Supplementary Fig. 7d, e). This may be due to the substantially faster overall migration of huBECs compared to mouse BECs. Importantly, the Axl inhibitor Bemcentinib^50^ did not impair huBEC motility (Supplementary Fig. 7e), demonstrating that TAM-mediated migration is predominantly driven by Mer in both huBECs and mouse BECs.

### Specific inactivation of endothelial Mer signaling in vivo

Our prior genetic analyses of Mer signaling in brain ECs were carried out with germline *Mertk* mouse knock-outs^12^. As noted above, Mer is also expressed by microglia^8,9^, tissue macrophages throughout the body^2^, and other cells^30^. We therefore crossed floxed *Mertk* alleles^8^ with a tamoxifen-inducible VE-cadherin Cre line, *Cdh5Cre*^*ER*^^51^, to generate mice in which we could conditionally inactivate *Mertk* in ECs while sparing microglia. We then assessed the performance of these mice in an established in vivo brain photothrombotic stroke model. In this model, mice are injected with the xanthene dye Rose Bengal (RB), and then subjected to brief (10 min) cold-light focal illumination of their brains (see Methods). RB illumination triggers the local formation of blood clots, leading immediately to cerebral stroke^52,53^.

*Cdh5Cre*^*ER*^*Mertk*^*f/f*^ mice (2–4 months) were injected IP on 5 successive days with either tamoxifen or vehicle (corn oil) control. Although the floxed *Mertk* allele and *Cdh5Cre*^*ER*^ driver have both been validated previously, we nonetheless verified that this protocol led to the sustained loss of endothelial Mer, by preparing BECs from mice 2 months after injections with vehicle or tamoxifen, and analyzing these cells for Mer expression and for the ability of Gas6 to stimulate Erk1/2 phosphorylation. We confirmed the long-term loss of active Mer (the floxed allele deletes a cassette exon within the Mer kinase domain^8^) and the inability of added Gas6 to stimulate Erk1/2 activation (Supplementary Fig. 8a, b). We further confirmed that expression of *Dll4*, *Nos3*, *Igfbp3*, and *Meox2* mRNAs was reduced in BECs prepared from tamoxifen-injected *Cdh5Cre*^*ER*^*Mertk*^*f/f*^ mice (Supplementary Fig. 8c) to an extent that paralleled the reductions seen in *Mertk*^*−/−*^ BECs (Fig. 2c). For our stroke studies, *Cdh5Cre*^*ER*^*Mertk*^*f/f*^ mice were injected IP with RB 15 days after their first vehicle or tamoxifen injection, and then immediately subjected to photothrombotic illumination of the brain (see Methods).

**Fig. 5:**
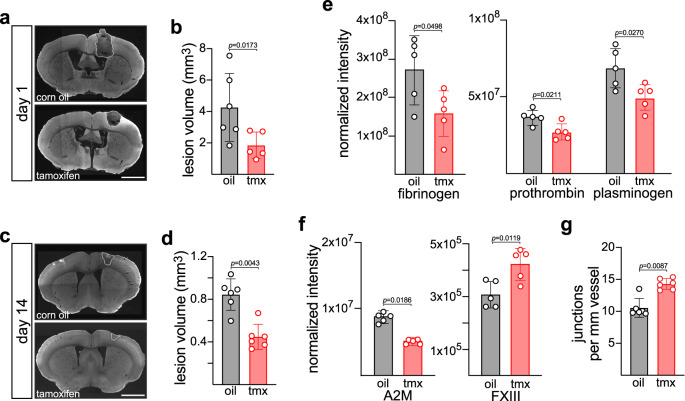
In vivo effects of conditional inactivation of the *Mertk* gene in ECs. **a** Representative examples of lesion volumes (white dotted areas) 1 d after the induction of blood-clot-produced photothrombotic stroke in corn oil-treated (control, top) versus tamoxifen-treated (*Mertk* deleted in ECs, bottom) *Cdh5Cre*^*ER*^*Mertk*^*f/f*^ mice (see Methods). **b** Quantification of results in (**a**). Each point is the lesion volume in an individual mouse as estimated by Cavalieri’s principle (see Methods) (**c**) Representative examples (left images) of lesion volumes (white dotted areas) 14 days after the induction of blood-clot-produced photothrombotic stroke in corn oil-treated (control, top) versus tamoxifen-treated (*Mertk* deleted in ECs, bottom) *Cdh5Cre*^*ER*^*Mertk*^*f/f*^ mice. **d** Quantification of results in (**c**). Each point is the lesion volume in an individual mouse as estimated by Cavalieri’s principle (see Methods). Plasma levels of fibrinogen, prothrombin and plasminogen (**e**), and α2-macroglobulin (A2M) and Factor XIII (**f**) in tamoxifen and corn oil-treated *Cdh5Cre*^*ER*^*Mertk*^*f/f*^ mice, as quantified by total proteome mass spectrometry. **g** Number of junctions formed per vessel in lesion area of corn oil-treated (control) versus tamoxifen-treated (*Mertk* deleted in ECs) *Cdh5Cre*^*ER*^*Mertk*^*f/f*^ mice (see Methods). Each data point represents the mean of an individual mouse (see Methods). Graphs show mean ±1 SD. Statistical significance was evaluated with a Mann Whitney test (**b**, **d**, **g**). Scale bars: **a**, **c** 2 mm.

Prior to any analyses of vessel structural perturbations, we found that the loss of Mer in ECs had an immediate and unanticipated effect on the size of the stroke lesion itself. At 1 day after illumination, the average lesion volume in corn oil control *Cdh5Cre*^*ER*^*Mertk*^*f/f*^ mice was ~4.2 mm^3^, but was only ~1.8 mm^3^ in tamoxifen-injected *Cdh5Cre*^*ER*^*Mertk*^*f/f*^ mice (Fig. 5a, b). This reduction was maintained throughout the full course of recovery after stroke, such that by 14 days after illumination, lesion volume averaged ~0.84 mm^3^ in the brains of control mice compared to ~0.45 mm^3^ in tamoxifen-injected mice (Fig. 5c, d).

What might account for this effect? We considered the possibility that EC Mer might normally bind and thereby lower the levels of circulating Pros1, since Pros1 is a Mer ligand^5^. In the absence of EC Mer, we hypothesized that plasma levels of Pros1, which is also an anticoagulant in the blood coagulation cascade^25^, might rise, resulting in reduced blood clotting and smaller strokes. However, ELISA measurements of plasma Pros1 levels in corn oil versus tamoxifen-injected *Cdh5Cre*^*ER*^*Mertk*^*f/f*^ mice revealed no difference (Supplementary Fig. 8d). As detailed above, proteomic analyses in WT versus *Mertk*^*−/−*^ BECs in culture showed that other coagulation factors were dysregulated in the mutant BECs (Supplementary Fig. 5a). While the procoagulants Factor V (F5) and Factor X (F10) were increased in *Mertk*^*−/−*^ BECs, the procoagulant von Willebrand factor (vWF) was decreased (Supplementary Fig. 5a). Much of the vWF in circulation is synthesized by ECs. Quantification of our BEC proteomic analyses revealed modest F5/10 expression, with modest increases in *Mertk*^*−/−*^ BECs relative to WT (Supplementary Fig. 8e). In contrast, vWF was highly expressed, and exhibited a ~2.5-fold drop in expression in *Mertk*^*−/−*^ BECs relative to WT (Supplementary Fig. 8e). Consistent with these protein results, our RNA-seq analyses showed that BEC *Vwf* mRNA levels were markedly reduced in *Mertk*^*−/−*^ BECs relative to WT, while those of *F5* and *F10* were not significantly regulated (Supplementary Fig. 8f).

Given these results, we performed a proteomic analysis of coagulation factors (and all other proteins) in plasma obtained from tamoxifen versus corn oil-injected *Cdh5Cre*^*ER*^*Mertk*^*f/f*^ mice using mass spectrometry. Although vWF protein and *Vwf* mRNA were both down-regulated in cultured *Mertk*^*−/−*^ BECs, vWF protein levels were unchanged in plasma from tamoxifen versus corn oil-injected *Cdh5Cre*^*ER*^*Mertk*^*f/f*^ mice. However, we detected multiple significant perturbations in the levels of proteins involved in the deposition, cross-linking, and metabolism of fibrin, the central constituent of blood clots. Most obviously, plasma fibrinogen, the precursor to fibrin, was reduced by ~40% in tamoxifen versus corn oil-injected *Cdh5Cre*^*ER*^*Mertk*^*f/f*^ mice (Fig. 5e). The precursor to thrombin (prothrombin), the serine protease that cleaves fibrinogen to produce fibrin, was also reduced by ~27%, and the precursor to plasmin (plasminogen), the serine protease that subsequently metabolizes fibrin clots, was reduced by ~29% (Fig. 5e). In addition, plasma expression of the plasmin regulator α2-macroglobulin (A2M) was reduced by ~45%, while those of the transglutaminase Factor XIII, which cross-links fibrin, were increased by ~30% (Fig. 5f). The pleiotropic regulation of these clotting factors in plasma from tamoxifen-treated *Cdh5Cre*^*ER*^*Mertk*^*f/f*^ mice may account for their milder strokes. Importantly, our observation of reduced stroke volumes upon endothelial inactivation of Mer signaling has striking parallels (discussed below) to reductions in stroke volume and blood clotting that have previously been reported when WT mice are treated with UNC2025 and then subjected to several thrombosis models^54^.

We also quantified multiple features of blood vessel structure and function 14 d after focal photothrombotic stroke in the presence or absence of endothelial Mer. The most significant perturbation we detected was a ~40% increase in new vascular branching/sprouting within the lesions of tamoxifen- versus corn oil-treated *Cdh5Cre*^*ER*^*Mertk*^*f/f*^ mice (Fig. 5g). This increase is predicted by the results documented above. During sprouting angiogenesis, vascular ECs undergo directed filopodial extension and migration of leading ECs, referred to as ‘tip’ cells, while following ‘stalk’ ECs proliferate and establish junctions that stabilize the newly formed vascular sprout^55^. Dll4/Notch signaling potently regulates tip cell formation and vessel density, and disruption of the Dll4 pathway in ECs results in increased tip cell formation and enhanced sprouting angiogenesis^34,56^. As documented above, *Dll4* mRNA was markedly down-regulated both by acute treatment of these cells with UNC2025 (Fig. 2a, c) and by *Mertk* mutation (Fig. 2a–c). Similarly, we found that mRNA encoding the Netrin-1 receptor Unc5 (Supplementary Fig. 4a) was markedly down-regulated both by acute treatment of these cells with UNC2025 and by *Mertk* mutation (Fig. 2a, c). Consistent with this dependence, mutations in the mouse *Unc5* gene have also been found to result in enhanced sprouting angiogenesis^36^.

We measured several additional features of vascular structure and function within both lesion sites and the ischemic border zone (IBZ) surrounding lesions in tamoxifen- versus corn oil-treated *Cdh5Cre*^*ER*^*Mertk*^*f/f*^ mice that had been subjected to photothrombotic stroke. While average vessel length (Supplementary Fig. 8g) and mean lacunarity (Supplementary Fig. 8h) both exhibited trends toward increases within tamoxifen-treated *Cdh5Cre*^*ER*^*Mertk*^*f/f*^ lesion sites, these differences were not statistically significant. However, mean IgG intensity within the IBZ - immunoglobulin from the circulation that had crossed a leaky BBB - was significantly higher in these mice (Supplementary Fig. 8i). This increase is very similar to the increase in IgG in the brain parenchyma that we previously observed in germline *Axl*^*−/−*^*Mertk*^*−/−*^ double knock-outs^12^, and argues that IgG leakage was due to the loss of TAM signaling in vascular ECs. As noted above, these in vivo effects are further consistent with the diminished expression of the essential BBB components perlecan, nidogen 1/2, Sparc, and laminins a4, a5, b1, b3, and c1 that we quantified in proteomic analyses of *Mertk*^*−/−*^ BECs relative to WT (Supplementary Fig. 5a). Together, these in vivo analyzes demonstrate that the EC-specific genetic inactivation of Mer leads to pleiotropic deficits in angiogenesis, BBB formation, and blood coagulation.

## Discussion

We define a new signal transduction pathway for the regulation of EC physiology. This pathway, which is controlled by the RTK Mer, regulates three key EC activities. The first is the organization of the EC cytoskeleton and basement membrane, the formation of tight junctions, and the maintenance of the BBB. Our earlier in vivo analyzes demonstrated that the *Mertk*^*−/−*^ BBB was leaky relative to WT^12^, and our new results demonstrate that this is also the case when Mer is selectively removed from ECs. This selective inactivation using independent floxed *Mertk*^*−/−*^ alleles is important, since the original *Mertk*^*−/−*^ mutation carries a segment of DNA from 129P2/Ola mice, which harbors a gene (or genes) that may modify the *Mertk*^*−/−*^ mutation^19,21^. (In the RPE, this gene has been shown to be the closely-related *Tyro3*^19^). It is therefore possible that other unknown genes might contribute to any phenotypes associated with the loss of Mertk in this original *Mertk*^*−/−*^ mutation. (Mouse ECs do not express Tyro3.) BBB leakiness is correlated with broad perturbations in the phosphorylation status and expression of cytoskeletal and basement membrane proteins in *Mertk*^*−/−*^ BECs and in WT BECs treated with a Mer tyrosine kinase inhibitor. Gas6 stimulated the rapid phosphorylation of multiple sites in Afap1, paracingulin, catenin alpha 2, and plectin, and these phosphorylation events were all reduced in *Mertk*^*−/−*^ BECs. Conversely, the phosphorylation of five regulatory sites in filamin A, three sites in filamin B, and six sites in filamin A-interacting protein 1l was enhanced in *Mertk*^*−/−*^ BECs. At the same time, the expression level of five different laminins—A4, A5, B1, B3, and C1—which are integral to the formation of the basement membrane, was reduced in these mutant ECs. These findings demonstrate that the Mer pathway coordinates the activity and expression of a large suite of proteins that are required to maintain the BBB.

The second Mer-regulated activity controls the expression of EC genes that encode regulators of angiogenesis. Mer activation elevates expression of proangiogenic genes (e.g., *Dll4* and *Unc5b*) and reduces expression of antiangiogenic genes (e.g., *Angpt2* and *Cited2*). This is again achieved via Mer-promoted phosphorylation of Akt, and the subsequent Akt-mediated phosphorylation of the downstream transducer Foxo1. Mer-mediated phosphorylation of Foxo1 drives it from the nucleus, where it normally represses proangiogenic and activates antiangiogenic genes. This general pathway also operates for VEGF and Tie2 receptor signaling systems in ECs. Even so, EC-specific inactivation of Mer alone, using *Cdh5Cre*^*ER*^*Mertk*^*f/f*^ mice, resulted in exuberant vascular branching after photothrombotic stroke.

Although the third activity that we find to be Mer-regulated—the plasma expression of fibrin-related proteins of the coagulation cascade—was unexpected, our finding that strokes are less severe upon loss of Mer signaling is not unprecedented. Just as we observed smaller photothrombotic stroke lesion volumes in mice in which *Mertk* was genetically ablated in ECs, earlier studies revealed reduced thrombosis, comparable in magnitude to those we describe, in WT mice treated with the UNC2025 Mer kinase inhibitor and then subjected to several different microvascular thrombosis protocols^54^. Although the ability of UNC2025 to reduce thrombosis relative to saline-treated controls was interpreted in the context of its inhibition of Mer in platelets, where this RTK is also expressed^54^, the drug of course inhibits Mer in all cells, including vascular ECs. Importantly, the effects of UNC2025 on platelet accumulation and α-granule secretion were modest relative to observed reductions in thrombosis^54^. This suggests that a significant locus of UNC2025 action with respect to its ability to reduce thrombosis may be the inhibition of Mer in vascular ECs. That notwithstanding, fibrinogen, prothrombin, plasminogen, and Factor XIII are overwhelmingly the products of hepatocytes in the liver, and so the dysregulation of their plasma expression upon EC-specific *Mertk* gene ablation must be cell non-autonomous. We have not identified a mechanism for this cell non-autonomous regulation.

The Mer regulation of blood clotting that we document may be relevant to disease settings beyond stroke. We suggest that this may be especially true for clotting pathologies that develop subsequent to infection with SARS-CoV-2, as EC/vascular dysfunction is now recognized as playing a central role in COVID-19 pathology^57,58^. Importantly, a recent proteomic analysis of blood plasma collected from a large cohort of patients with mild, moderate, or severe COVID-19 revealed that the single most informative plasma feature for disease severity was soluble Mer (sMer), the levels of which were found to increase with increasingly severe disease^59^. This rise in plasma sMer was not associated with the elevation of other monocyte activation markers, suggesting that it was derived from Mer activation in cells other than macrophages. sMer is generated by protease cleavage of the receptor ectodomain from full-length Mer, and is an invariant consequence of Mer activation^60^. Our data suggest that high levels of vascular Mer signaling during COVID-19 infections, which would obligatorily result in high levels of sMer in plasma, would also lead to elevated fibrin deposition. Blood levels of the so-called “D-dimer” of fibrin, a proteolytic fragment that is a clinically measured indicator of blood clotting, have also proven to be an excellent predictor of COVID-19 severity^57,58^.

Together, the above results indicate that the Mer signaling pathway is an important regulator of EC physiology in the mature vasculature. Mer, Axl and Tyro 3 are currently being targeted—using antibody inhibitors, ligand traps, and small-molecule inhibitors of the TAM kinases—in the context of cancer^61,62^. To our knowledge, the effects that these potential therapeutic inhibitors might have on endothelial cell function have not been considered.

## Methods

### Mice

All mice were bred and housed at the Salk Institute Animal Facility under a 12 h light/dark cycle in a pathogen-free environment. *Mertk*^−/−^^13^, *Mertk*^*f/ f*^^8^, *Axl*^−/−^^13^, *Gas6*^−/−^^63^, *Pros1*^*f/ f*^^25^, and *Cdh5Cre*^*ER*^ ^51^ mice have been described previously. All mice were on a pure C57Bl/6 background. All experiments and procedures were conducted according to guidelines established by the Institutional Animal Care and Use Committee (IACUC).

### BEC isolation and culture

BECs were isolated from the cortices of 8–12 week mice (8–10 mice per genotype per culture) as described previously^18^, and cultured on plates coated with 100 μg/ml collagen IV (Sigma) and 100 μg/ml Fibronectin (Sigma). Cultures were maintained in DMEM (Gibco) supplemented with Penicillin–Streptomycin (Gibco), 20% heat inactivated heat-inactivated fetal bovine serum (FBS, Sigma) and 1 ng/ml basic fibroblast growth factor (bFGF, Invitrogen). The medium was supplemented with 3 μg/ml puromycin (Sigma) for the first 3 days to selectively propagate puromycin-resistant BECs. Human BECs (Cell Systems) were cultured on plates coated with 150 μg/ml rat tail collagen I (EMD Millipore) in EBM2 basal medium (Lonza) supplemented with Penicillin-Streptomycin, 5% FBS, 1.4 μM hydrocortisone (Sigma), 5 μg/ml ascorbic acid (Sigma), 1 ng/ml bFGF and 10 mM Hepes (Sigma).

### Reagents

Mouse and human Gas6 were expressed and purified as described previously^5,16^. Mouse VEGF-A was from Life Technologies. The Mer inhibitor UNC2025, the Axl inhibitor Bemcentinib, and the Akt inhibitor Miransertib were all from Selleck Chemical LLC. A list of antibodies and primers used can be found in Supplementary Table 1.

### Scratch assay

Confluent BEC or huBEC monolayers were treated ±300 nM UNC2025 for 30 min, after which a scratch was induced through the center of the well using a pipet tip. Cells were rinsed three times and serum-free medium supplemented with 10 nM Gas6 ±300 nM UNC2025, as indicated, was added. Cells were imaged at time points 0 h and at 5 h (human BECs) or 0 h and 18 h (mouse BECs) and % gap closure was measured in Fiji.

### Transwell migration assay

Transwell inserts with a 5 μm pore size (Corning) were coated on both sides with 100 μg/ml collagen IV and 100 µg/ml fibronectin, and 60,000 BECs in serum-free medium supplemented with 300 nM UNC2025, as indicated, were added to the upper chamber. Serum-free medium with 10 nM Gas6 and/or 300 nM UNC2025 was added to the bottom chamber, and cells were allowed to migrate for 18 h. Cells remaining on the top of the membrane were removed using a cotton swab and inserts were rinsed with PBS. Cells that had migrated to the bottom side of the membrane were stained and fixed for 20 min in crystal violet solution (0.05% Crystal Violet, 1% formaldehyde and 1% Methanol in PBS), after which membranes were imaged. For quantification, dye was extracted for 10 min in 100 μl 10% acetic acid and supernatant absorbance was measured at 590 nm.

### Tube formation assay

Plates (24-well) were coated with 250 μl Geltrex LDEV-free reduced growth factor basement membrane matrix (ThermoFisher) and 100,000 BECs were seeded on top of the matrix in serum-free medium supplemented with 10 nM Gas6 and 300 nM UNC2025, as indicated. Wells were imaged at 5 h post-seeding and tube formation was quantified using Fiji software with the Angiogenesis Analyzer plugin.

### Bulk RNA seq on mouse BECs

WT and *Mertk*^*−/−*^ mouse BECs were isolated as described above and cultured until confluent. Cells were incubated for 24 h in the presence or absence of 300 nM UNC2025, after which time total RNA was isolated using the NucleoSpin RNA kit (Macherey-Nagel). Quality of the isolated RNA was assessed using Agilent TapeStation 4200 and RNA-Seq libraries were prepared with 500 ng total RNA using the TruSeq stranded mRNA Sample Preparation Kit according to the manufacturer’s protocol (Illumina). RNA-seq libraries were multiplexed, normalized, and pooled for sequencing. The libraries were sequenced on the HiSeq 2500 system (Illumina) at single read 50 bp. Image analysis and base calling were done with Illumina CASAVA-1.8.2. on HiSeq 2500 system and sequenced reads were quality-tested using FASTQC. The obtained reads were mapped to the mm10 genome using STAR v2.5.3a^64^ and gene expression quantified with HOMER v 4.10.4^65^ using the fragments per kilobase per million mapped reads (FPKM) normalization across exons of the top isoform. Heatmaps show z-score normalized relative expression across conditions for each gene.

### RT-qPCR

RNA was harvested from cultured BECs using the NucleoSpin RNA kit (Macherey-Nagel). Reverse transcription was performed with the Transcriptor First Strand cDNA Synthesis kit (Roche) with anchored oligo(dT) primers. Quantitative PCR was run using SYBR Green PCR Master mix (Applied Biosystems) on a QuantStudio 5 (Applied Biosystems). A list of the primers used can be found in the supplementary Table 1.

### Total proteome and phosphoproteome mass spectrometry

For phosphoproteomic analyzes, WT and *Mertk*^*−/−*^ BECs were incubated in medium with 1% FBS o/n followed by 2 h in serum-free medium. Cells were then stimulated with 10 nM Gas6 for 15 min, after which cells were lysed in RIPA buffer (50 mM Tris-HCl, pH 8.0, 150 mM NaCl, 0.1% SDS, 1% Triton X-100, 0.5% deoxycholate) supplemented with HALT protease and phosphatase inhibitor cocktail (Thermo Fisher) and 1 mM sodium orthovanadate. Lysates were centrifuged at 10,000 rpm for 3 min at 4 °C (TOMY MRX-150) and a BCA assay (Thermo Fisher) was performed on the supernatants to measure protein concentration. For total proteome analysis, lysates were prepared as above from confluent cultures of WT and *Mertk*^*−/−*^ BECs grown in full medium, and plasma samples were collected by cardiac puncture as described below. Samples were precipitated using methanol-chloroform. Dried pellets were dissolved in 8 M urea/100 mM TEAB, pH 8.5, reduced with 5 mM tris(2-carboxyethyl) phosphine hydrochloride, and alkylated with 50 mM chloroacetamide. Proteins were then trypsin digested overnight at 37 °C. The digested peptides were labeled with TMT (Thermo product 90309, lot UB274629, XB318561), and lysate and plasma samples were fractionated by basic reversed phase (Thermo 84868). Phosphopeptide samples were enriched by Fe-NTA (Thermo A32992). The TMT labeled samples were analyzed on a Fusion Lumos mass spectrometer (Thermo). Samples were injected directly onto a 25 cm, 100 μm ID column packed with BEH 1.7 μm C18 resin (Waters). Samples were separated at a flow rate of 300 nl/min on a nLC 1200 (Thermo). Buffer A and B were 0.1% formic acid in water and 90% acetonitrile, respectively. A gradient of 1–25% B over 180 min, an increase to 40% B over 30 min, an increase to 100% B over another 20 min and held at 90% B for a 10 min was used for a 240 min total run time for the fractionated lysate samples. Phospho-enriched samples were analyzed by three injections (unfractionated) with a 120 min RP gradient as follows: of 1–25% B over 75 min, an increase to 40% B over 30 min, an increase to 100% B over another 10 min and held at 90% B for 5 min. Protein samples were analyzed on an Orbitrap Eclipse mass spectrometer in a similar manner. A gradient of 1–25% B over 120 min, an increase to 40% B over 40 min, an increase to 100% B over another 10 min, and a hold at 90% B for 10 min was used for a 180 min total run time. The column was re-equilibrated with 15 μl of buffer A prior to the injection of sample. Peptides were eluted directly from the tip of the column and nanosprayed directly into the mass spectrometer by application of 2.8 kV voltage at the back of the column. The Lumos and Eclipse were operated in a data dependent mode. Full MS1 scans were collected in the Orbitrap at 120k resolution. The cycle time was set to 3 s, and within this 3 s the most abundant ions per scan were selected for CID MS/MS in the ion trap. MS3 analysis with multinotch isolation (SPS3) was utilized for detection of TMT reporter ions at 60k resolution^66^. Monoisotopic precursor selection was enabled and dynamic exclusion was used with exclusion duration of 10 s.

Protein and peptide identification were done with Integrated Proteomics Pipeline—IP2. Tandem mass spectra were extracted from raw files using RawConverter^67^ and searched with ProLuCID^68^ against Swiss-Prot mouse database. The search space included all fully-tryptic and half-tryptic peptide candidates. Carbamidomethylation on cysteine and TMT labels on N terminus and lysine were considered as static modifications. Phosphorylation was considered as a variable modification on STY Data was searched with 50 ppm precursor ion tolerance and 600 ppm fragment ion tolerance. Identified proteins were filtered to 10 ppm precursor ion tolerance using DTASelect^69^, with a target-decoy database search strategy to control the false discovery rate to 1% at the protein level^70^. Quantitative analysis of TMT was done with Census^71^, filtering reporter ions with 20 ppm mass tolerance and 0.6 isobaric purity filter.

### Western blot

WT and *Mertk*^*−/−*^ BECs or huBECs were incubated in medium with 1% FBS (mouse BECs) or 0.5% FBS (huBECs) o/n followed by 2 h in serum-free medium. When evaluating the effect of kinase inhibitors on signaling, the inhibitors were supplemented to the serum-free medium for the last 30 min of the incubation, at concentrations indicated. Cells were then stimulated with 10 nM Gas6 for 15 min in the presence or absence of inhibitors, after which cells were lysed in RIPA buffer supplemented with HALT protease and phosphatase inhibitor cocktail and 1 mM sodium orthovanadate. Lysates were centrifuged at 10,000 rpm for 3 min at 4 °C (TOMY MRX-150) and a BCA assay (Thermo Fisher) was performed on the supernatants to measure protein concentration. Proteins (5–20 μg total lysate) were separated on 4–12% Bis Tris Plus gels (Life Technologies) and transferred to PVDF membranes. Membranes were blocked in PBS with 1% Casein (BioRad) and incubated with primary antibodies diluted in blocking buffer o/n at 4 °C. Membranes were washed three times with TBS supplemented with 0.1% Tween (TBST), and incubated for 2 h with secondary HRP-conjugated antibodies in blocking buffer. After three washes with TBST, the membranes were developed using the SuperSignal West Pico PLUS Chemiluminescent substrate (Thermo Scientific).

### Foxo1 cytoplasmic translocation

WT BECs were grown to 80% confluence on Ibidi eight-well μ-slides coated with 100 μg/ml collagen IV and 100 μg/ml fibronectin. Cells were incubated in serum-free medium for 2 h, after which 10 nM Gas6 was added for 30 min. Cells were washed with PBS, fixed in 4% PFA in PBS and permeabilized with 0.5% Triton X-100. Free aldehyde groups were blocked with 0.12% glycine in PBS after which cells were incubated with 1% BSA in PBS for 15 min. Foxo1 was visualized using anti-Foxo1 followed by Alexa Fluor 488-conjugated secondary antibodies. Hoechst was added to the secondary antibody for nuclear staining. Cells were washed and immediately imaged with a Zeiss LSM 710 Confocal microscope using Plan-Apochromat 20 × 0.8-NA air-matched objective. Colocalization of Foxo1 and Hoechst was calculated using the JACoP plugin in Fiji^72^, based on imaging of a minimum of 1200 cells per condition over three independent experiments.

### Immunohistochemistry for Mer

Adult (9 months) *WT* mice were anesthetized with 100 mg/kg Ketamine and 16 mg/kg Xylazine and perfused transcardially with 20 U/ml heparin in PBS followed by 4% paraformaldehyde in PBS. Brains were harvested and both hemispheres were post-fixed o/n at 4 °C in 4% paraformaldehyde in PBS. Brains were incubated in 30% sucrose in PBS for 24 h and frozen in TBS Tissue Freezing Medium (TFM, Electron Microscopy Sciences). Brains were sagittally cryosectioned at 17 microns and air-dried o/n. Heat mediated antigen retrieval was performed in 10 mM citrate buffer pH 6.0 for 3 min at 80 °C and slides were allowed to cool down to RT in the same buffer. Non-specific binding was prevented by incubating sections in 0.12% glycine solution for 30 min at RT, followed by a 1 h incubation in blocking buffer (PBS containing 0.3% Triton-X100, 0.1% Tween-20, 5% donkey serum and 2% IgG-free BSA). Sections were incubated with primary antibodies diluted in blocking buffer o/n at 4 °C, washed three times with PBS containing 0.1% Tween-20 and incubated for 2 h at RT with Alexa Fluor conjugated secondary antibodies together with Hoechst in blocking buffer. Sections were washed as above and sealed with Fluoromount G (SouthernBiotech).

### Capillary density measurement

Adult WT (*n* = 4) and *Mertk*^*−/−*^ (*n* = 4) mice at 6mo, were anesthetized with 100 mg/kg Ketamine and 16 mg/kg Xylazine in accordance with IACUC guidelines, whereupon they received a retro-orbital injection of 50 μg DyLight649-conjugated tomato lectin (Vector Laboratories). The dye was allowed to circulate for 5 min, after which the mice were euthanized by cervical dislocation, and the brains harvested. The brains were immediately frozen in Tissue-Tek O.C.T. Compound (Sakura). Brains were sagittally cryosectioned at 30 μm and 8 sections per brain, 120 μm apart, were mounted for analysis. Brains were stained with Hoechst diluted in PBS containing 0.3% Triton-X100, 0.1% Tween-20, 5% donkey serum and 2% IgG-free BSA, and sealed with Fluoromount-G. Tile scans of the prefrontal and the somatosensory cortex were obtained with an Olympus-VS120 Virtual Slide Scanning Microscope using a 10× objective. Capillary area in the obtained images was measured using AngioTool^73^.

### Photothrombotic stroke model

*Cdh5*Cre^ER^*Mertk*^*f/f*^ mice (2–4 months) were injected i.p. with 100 mg/kg tamoxifen (Sigma) dissolved in corn oil, or corn oil alone, for five consecutive days starting 15 days prior to photothrombosis. To induce a thrombotic lesion, mice received an i.p injection of 80 mg/kg Rose Bengal (Sigma) dissolved in PBS and 10 min later they were anesthetized using isoflurane (2.75% for induction, 1.5% for maintenance). Upon securing the mouse in a stereotaxic frame, the head was shaved and the skin sanitized, after which an incision was made along the midline to expose the skull. A cold light source (Zeiss KL 1500 LCD) with a flexible light guide connected to a 40× objective to focus the light, was directed ~1.5 mm lateral and 1 mm anterior to the bregma, and the skull was illuminated for 10 min at 3300 K. The skin was subsequently closed with 5–0 nylon sutures and Vetbond tissue adhesive, and animals were administered 0.5 mg/kg Buprenorphine SR as analgesics. Brains were harvested 1 or 14 days after photothrombosis. Mice were anesthetized with 100 mg/kg Ketamine and 16 mg/kg Xylazine, and perfused transcardially with 20 U/ml heparin in PBS followed by 4% paraformaldehyde in PBS. Brains were harvested and post-fixed o/n at 4 °C in 4% paraformaldehyde in PBS, incubated in 30% sucrose in PBS for 24 h and frozen in TBS Tissue Freezing Medium (TFM, Electron Microscopy Sciences), after which they were coronally cryosectioned at 30 μm.

### Analysis of PHTH lesions

Immunohistochemistry on collected sections was performed as described above, with antigen retrieval in 10 mM citrate buffer pH 6.0 for 3 min at 80 °C. Images were obtained with an Olympus-VS120 Virtual Slide Scanning Microscope using a 10× objective. All analysis was performed in the CellProfiler software (version 4.2.1, Broad Institute)^74^. The lesion and the ischemic border zone 300 μm distal to the lesion were determined based on changes in MAP2 staining. For quantification of vascular parameters, images were individually thresholded, and where applicable, binarized. Noise was removed from binarized images by excluding particles below a size threshold of 50. Total vascular area, vascular branching, and tissue IgG was calculated based on CD31^+^ or IgG^+^ area in 4 sections per mouse, 120 μm apart, in the center of the lesion. Vascular branching in the lesion was measured using AngioTool, as above. Lesion volume was estimated based on Cavalieri’s volume estimate in sections 120 μm apart spanning the entire lesion.

### Plasma collection from *Cdh5Cre*^*ER*^*Mertk*^*f/f*^ mice

*Cdh5Cre*^*ER*^*Mertk*^*f/f*^ mice (2–4 months) were injected i.p. with 100 mg/kg tamoxifen (Sigma) dissolved in corn oil, or corn oil alone, for five consecutive days. Two weeks after the first injection, plasma was harvested. Mice were anesthetized with 100 mg/kg Ketamine and 16 mg/kg Xylazine, and blood was collected from the right ventricle using a 23 G needle into syringes containing 3.8% sodium citrate, for a final citrate to plasma ratio of 1:9. The blood was immediately transferred to an Eppendorf tube and gently inverted, after which it was centrifuged at 2500 g for 10 min at RT. The plasma was aliquoted and immediately stored at −80 °C. The plasma was subsequently used for Pros1 ELISA and MS analysis of total plasma proteome.

### ELISA for Mer, Axl, and Protein S

Mer and Axl were measured using Mouse Mer and Mouse Axl DuoSet ELISA (R&D Systems). To measure Protein S in plasma, polystyrene High Bind Microplates (Corning) were coated with a polyclonal anti-human Pros1 antibody (Dako) in 75 mM Na-carbonate pH 9.6 o/n at +4 °C. In between each assay step, plates were washed 3× with PBS containing 0.1% Tween 20. Wells were blocked with 2 % BSA in PBS, which was also used as sample and antibody dilution buffer. A standard curve was generated with a pooled plasma sample from 11 mice, and individual samples diluted at 1:400 were analyzed. Samples and standards were incubated on the plate for 2 h at RT, followed by incubation with a rat anti-mouse Pros1 antibody (R&D Systems) for 1 h. After an additional 1 h incubation with a goat anti-rat HRP conjugate (Jackson ImmunoResearch), the plates were developed with a TMB substrate (R&D Systems).

### Statistics and reproducibility

Statistical calculations were performed in GraphPad Prism version 8.0. All data represent the mean and SD of at least three individual experiments unless specified otherwise. The number of mice in each experimental group is indicated in the figure legends and plotted as individual data points in the graphs.

### Reporting summary

Further information on research design is available in the Nature Portfolio Reporting Summary linked to this article.

### Supplementary information

Supplementary Information

Description of Additional Supplementary Files

Supplementary Data 1

Reporting Summary

## Data Availability

RNAseq data have been deposited under GEO dataset accession number GSE225474, and are fully accessible. MS data were submitted to MassIVE with the dataset number MSV000091355, and are fully accessible. Uncropped and unedited blot images for all figures are provided in Supplementary Fig. 10. Source data for all graphs in the manuscript can be found in the source data file (Supplementary Data 1).
