# Supplementary material for: Regulation of brain endothelial cell physiology by the TAM receptor tyrosine kinase Mer
Source: Commun Biol. 2023 Sep 7;6:916. doi: 10.1038/s42003-023-05287-y (PMC10482977; doi:10.1038/s42003-023-05287-y)
Supplement: Supplementary file 1 — Supplementary Information [file 42003_2023_5287_MOESM1_ESM.pdf]

Supplementary Information

Regulation of brain endothelial cell physiology by the TAM receptor tyrosine kinase Mer

Kaisa E. Happonen, Patrick G. Burrola, and Greg Lemke

Molecular Neurobiology Laboratory

The Salk Institute for Biological Studies

La Jolla, CA 92037

Supplementary Table 1

Antibodies used in the study

Mouse	Cat nr	Source	Western blot	IHC/ICC	ELISA
Akt (pan)	4691	Cell Signaling	1:1000		
p-Akt (S473)	4058	Cell Signaling	1:1000		
Akt1s1	2691	Cell Signaling	1:1000		
p-Akt1s1 (T246)	2997	Cell Signaling	1:1000		
Axl	AF854	R&D systems	1:1000		
CD31	AF3628	R&D systems		1:200	
eNOS	32027	Cell Signaling	1:1000		
p-eNOS (S1177)	9571	Cell Signaling	1:1000		
Foxo1	2880	Cell Signaling	1:1000	1:100	
p-Foxo1 (S256)	84192	Cell Signaling	1:1000		
Iba1	019-19741	Wako			
Map2	ab5392	Abcam		1:10 000	
Mer	DS5MMR	eBiosciences	1:2000		
Tyro3	5585	Cell Signaling	1:500		
Protein S	MAB4976	R&D systems			1:500
P44/42	4695	Cell Signaling	1:1000		
p-P44/42 (T202/Y204)	9101	Cell Signaling	1:1000		
**Human**					
Akt (pan)	4691	Cell Signaling	1:1000		
p-Akt (S473)	4058	Cell Signaling	1:1000		
Axl	H-124	Santa Cruz	1:1000		
eNOS	32027	Cell Signaling	1:1000		
p-eNOS (S1177)	9570	Cell Signaling	1:1000		
Foxo1	2880	Cell Signaling	1:1000		
p-Foxo1 (S256)	84192	Cell Signaling	1:1000		
Mer	ab52968	Abcam	1:2000		
Protein S	A0384	Dako			10 μg/ml
P44/42	4695	Cell Signaling	1:1000		
P-p44/42 (T202/Y204)	9101	Cell Signaling	1:1000		

Primers used in the study

**Mouse**	**forward**	**reverse**
Adamts1	GAAGGCAAACGAGTCCGCTACA	TTGGGTGTCCACTCTACAGTGG
Adamts9	TACCGAGAACCCAGTGGCGATT	ACAGGCACTCTCATCAGCCACA
Angpt2	AACTCGCTCCTTCAGAAGCAGC	TTCCGCACAGTCTCTGAAGGTG
ApoE	GAACCGCTTCTGGGATTACCTG	GCCTTTACTTCCGTCATAGTGTC
Axl	AGGCTCATTGGCGTCTGTTTT	CTGTGTAGGTCTCCGTGTTTCATG
Ccbe1	GTTCTCCCAGATGAAGCAGACC	TCCTGGAAGGTAGGCGTTTGAG
Cited2	TGCCGCCCAATGTCATAGACAC	AGAGTTCGGGCAGCTCCTTGAT
Dll4	TGTCTCCACGCCGGTATTG	AGGTCGTCTCCCGGTGTGT
Ephb1	TGGGTGGGAAGAAGTCAGTG	AAAGGTGGTAAGCAGCCAG
Gas6	AACTGGCTGAACGGGGAAG	CTTCCCAGGTGGTTTCCGT
Igfbp3	GTGACCGATTCCAAGTTCC	CTTCAGATGATTCAGTGTGTCC
Itgb4	ACACAAGCTCCAGCAGACGAAG	TCCACCTGCTTCTCTGTCAGCT
Meox2	ACCACCACCATCACCATCAC	CGCTTTTCCTTTTGCTGCC
Mertk	CAGCTCGAAACTGCATGTTGC	GCAATGCGGCCTTGGCGGTA
Mertk ex18	GTGTGGGCTTTTGGCGTGA	GTCATACAGTTCATCCAAGCAG
Nos3	CACGTGCACAGGCGGAAGATGTTC	CTCCTGGCCTCCGGTGTCCAG
ProS1	GCACAGTGCCCTTTGCCT	CAAATACCACAATATCCTGAGACGTT
Sema3g	AACCGCTCTGCCATCTTTC	ACACATTCCACCTTCTGTCC
Unc5b	GGACAGTTACCACAACCTACGC	CTGCCATTCCAGACGTGGTAGA
**Human**		
Adamts1	GCGTCAATGCTTTCCAACCTGG	GGGATTCTGAGGCTTGTCCATC
Axl	GTTTGGAGCTGTGATGGAAGGC	CGCTTCACTCAGGAAATCCTCC
Cited2	TGCCGCCCAATGTCATAGACAC	CAGCTCCTTGATGCGGTCCAAA
Dll4	CTGCGAGAAGAAAGTGGACAGG	ACAGTCGCTGACGTGGAGTTCA
Ephb1	GCACCAAGTCAGTGCCACTATG	CCTTGCTGTGTTGGTCTGACTC
Gapdh	GTCTCCTCTGACTTCAACAGCG	ACCACCCTGTTGCTGTAGCCAA
Igfbp3	CGCTACAAAGTTGACTACGAGTC	GTCTTCCATTTCTCTACGGCAGG
Itgb4	AGGATGACGACGAGAAGCAGCT	ACCGAGAACTCAGGCTGCTCAA
Mertk	CAGGAAGATGGGACCTCTCTGA	GGCTGAAGTCTTTCATGCACGC
Nos3	GAAGGCGACAATCCTGTATGGC	TGTTCGAGGGACACCACGTCAT
Sema3g	CTGAGGAAGTGGTTCTGGAGGA	GCCGTAAGTCTCACATTGGTGC

Supplementary Figures

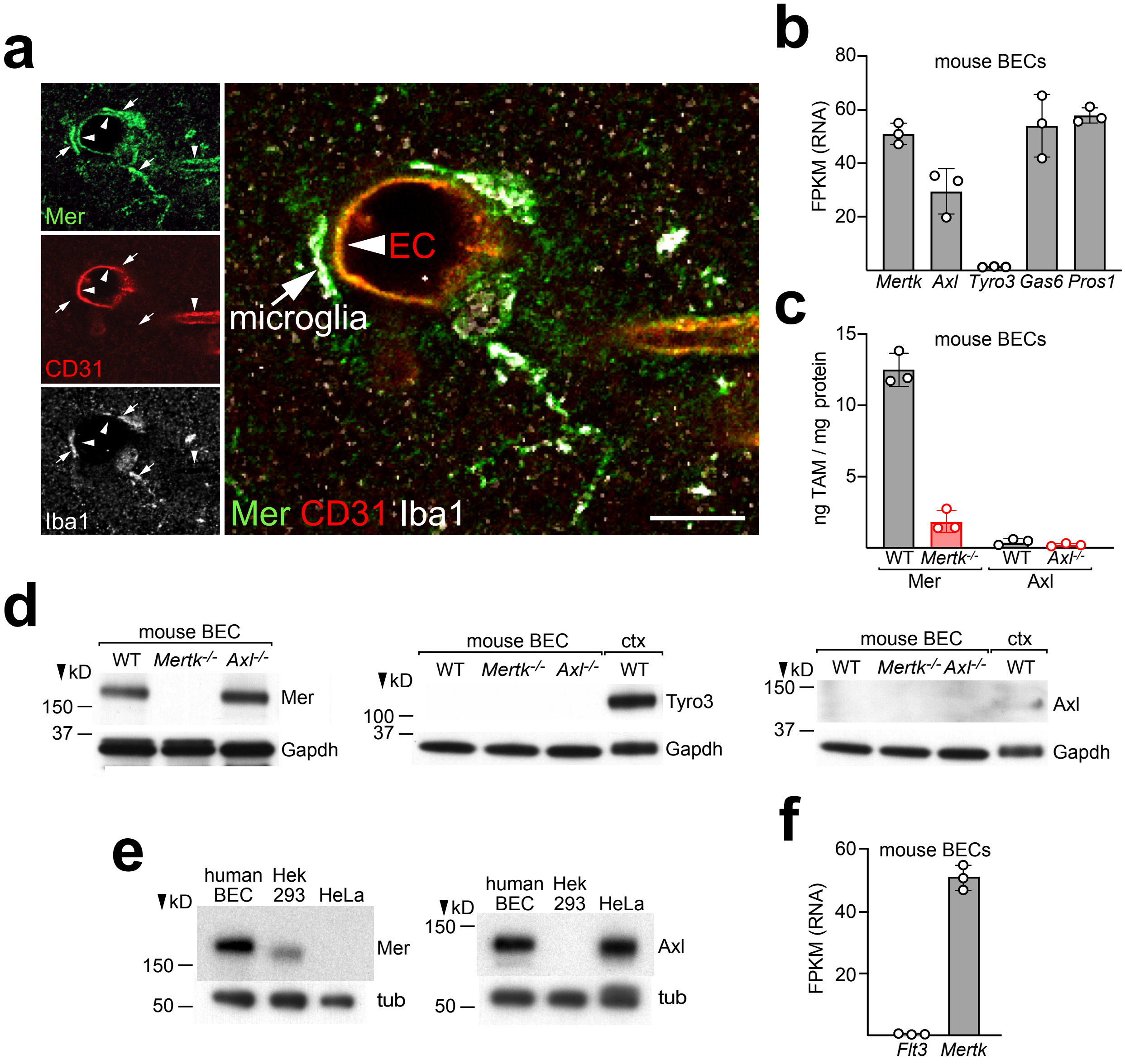

Supplementary Fig. 1. TAM receptor expression in mouse and human BECs. (**a**) Immunohistochemical localization of Mer (left top, green), the EC marker CD31 (left middle, red), and the microglial marker Iba1 (left bottom, white) in sections of adult mouse brain. Enlarged merged image is at right. Scale bar 10 μm. (**b**) TAM receptor and ligand mRNA levels in WT mouse BECs, as determined by RNA-seq (see Methods). (**c**) Mer and Axl protein levels in WT and *Mertk*$^{-/-}$ mouse BECs, as determined by ELISA (see Methods). Graphs show mean +/- 1 SD, and data points are from cell

lysates prepared from separate batches of isolated BECs. (**d**) Representative western blots of extracts of mouse BECs or mouse cortex (ctx), from mice of the indicated genotypes, probed with antibodies to Mer (left), Tyro3 (middle), or Axl (right). (**e**) Representative western blots of extracts of human BECs or the human cell lines Hek293 and HeLa probed with antibodies to Mer (left) or Axl (right). (**f**) Expression of Flt3 versus Mertk mRNA in mouse BECs, as determined by RNA-seq. Additional quantification of western blots in this figure, and blots in subsequent figures, is provided in Supplementary Fig. 9.

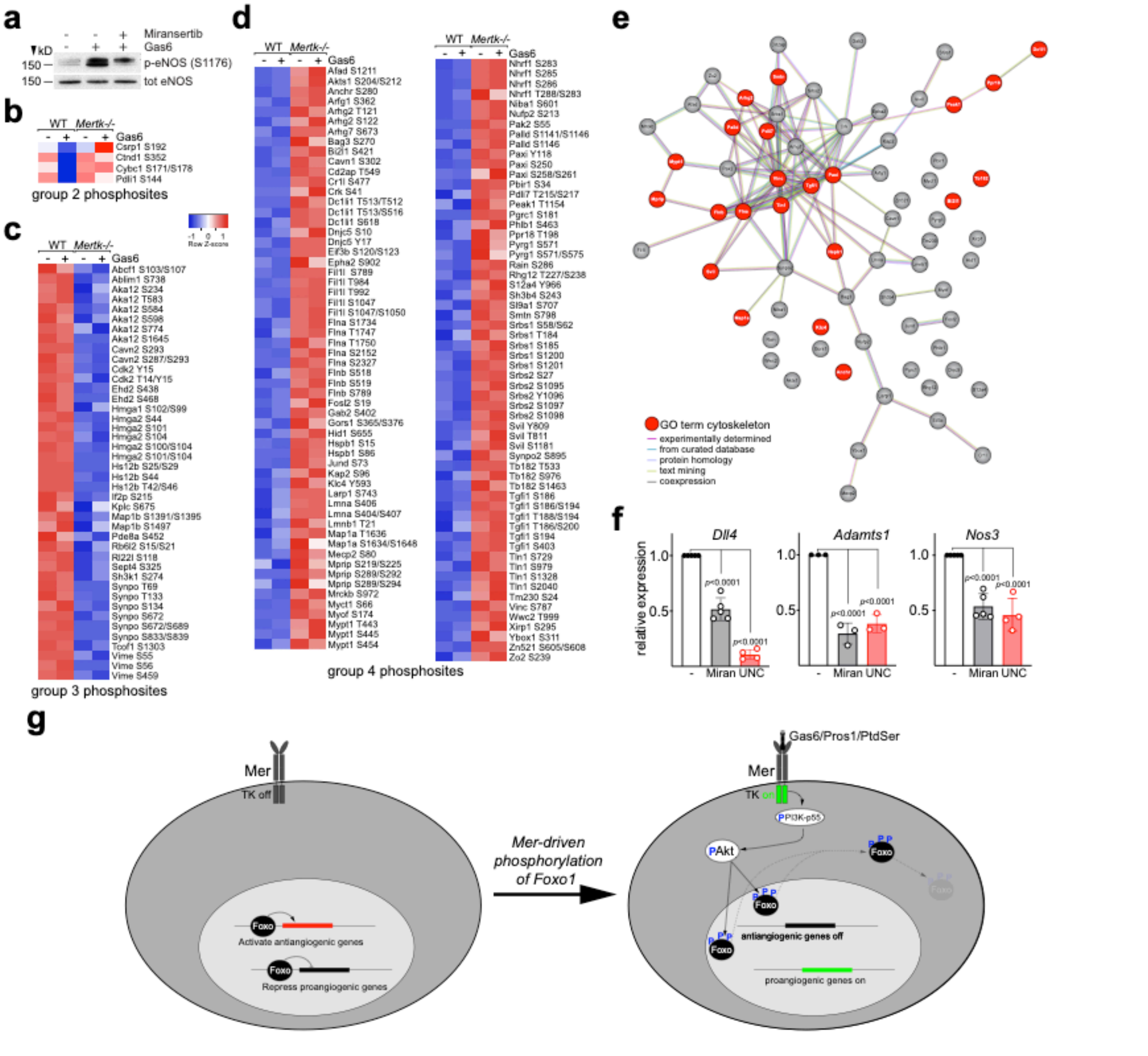

Supplementary Fig. 2. Mer regulation of phosphosite expression in mouse BECs.

(**a**) Representative western blot of extracts of mouse BECs treated with Gas6 (10 nM)

for 15 min, with and without prior incubation (30 min) with the Akt inhibitor Miransertib (100 nM), and probed with antibodies to phospho-eNOS (S1176) or total eNOS. (**b**) Heat maps of selected group 2 phosphosites whose expression was inhibited in WT mouse BECs by 15 min incubation with 10 nM Gas6, but not in *Mertk*$^{-/-}$ BECs. (**c**) Heat maps of selected group 3 phosphosites whose expression was not regulated in WT or *Mertk*$^{-/-}$ mouse BECs by 15 min incubation with 10 nM Gas6, but was constantly reduced in *Mertk*$^{-/-}$ BECs. (**d**) Heat maps of selected group 3 phosphosites whose expression was not regulated in WT or *Mertk*$^{-/-}$ mouse BECs by 15 min incubation with 10 nM Gas6, but was constantly increased in *Mertk*$^{-/-}$ BECs. (**e**) STRING functional association network analysis of group 4 phosphosites. Proteins associated with the gene ontology (GO) term cytoskeleton are highlighted in red. (**f**) Expression of the indicated mRNAs in mouse BECs treated with Miransertib (100 nM, 20 h) or UNC2025 (300 nM, 20 h) relative to untreated cells, as determined by qRT-PCR. Graphs show mean +/- 1 SD, and data points indicate experiments performed on lysates from 3 separate BEC isolations. Statistical significance of differences was evaluated with a one-way ANOVA using a Dunnett's post test. (**g**) A model of the differential effects of Mer-mediated Foxo1 phosphorylation on the expression of pro- versus antiangiogenic genes.

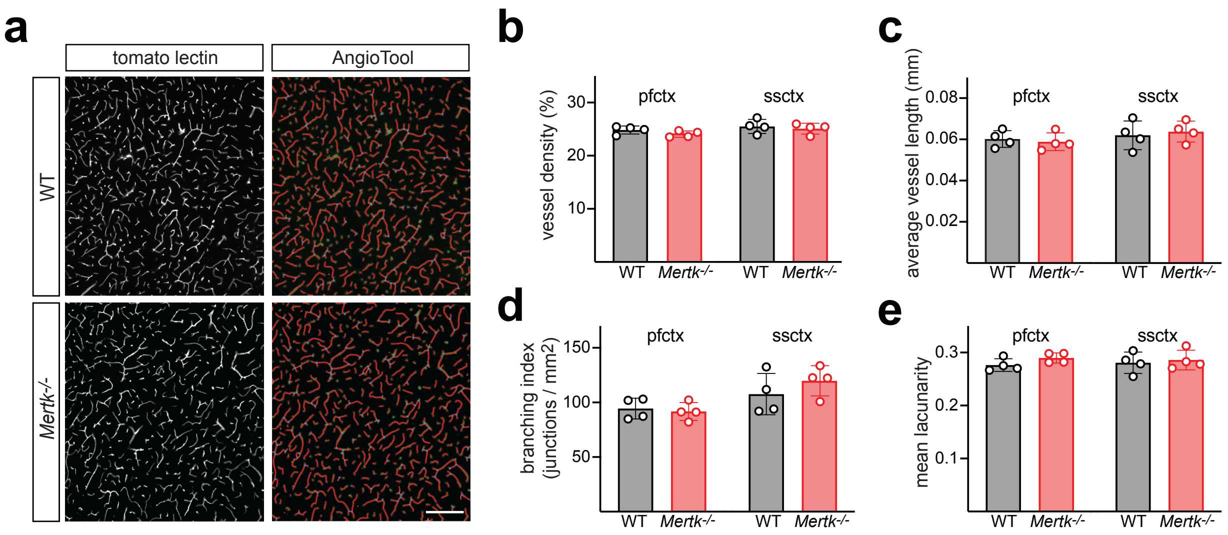

Supplementary Fig. 3. No perturbation of any feature of vascular structure in the adult brains of *Mertk*$^{-/-}$ mice. (**a**) Representative staining of the adult brain vasculature following IV injection of Lycopersicon esculentum agglutinin (tomato lectin), in WT (top) and *Mertk*$^{-/-}$ (bottom) mice; raw (left) and Angio Tool (right) analyzed images. (**b**) Vessel density; (**c**) Average vessel length; (**d**) Branching junctions; and (**e**) Lacunarity in prefrontal (pfctx) and somatosensory cortex (ssctx) in 4 separate WT and *Mertk*$^{-/-}$ mice. Graphs show mean +/- 1 SD, and statistical significance of differences was evaluated with a Mann Whitney test (panels b, c, d, and e).

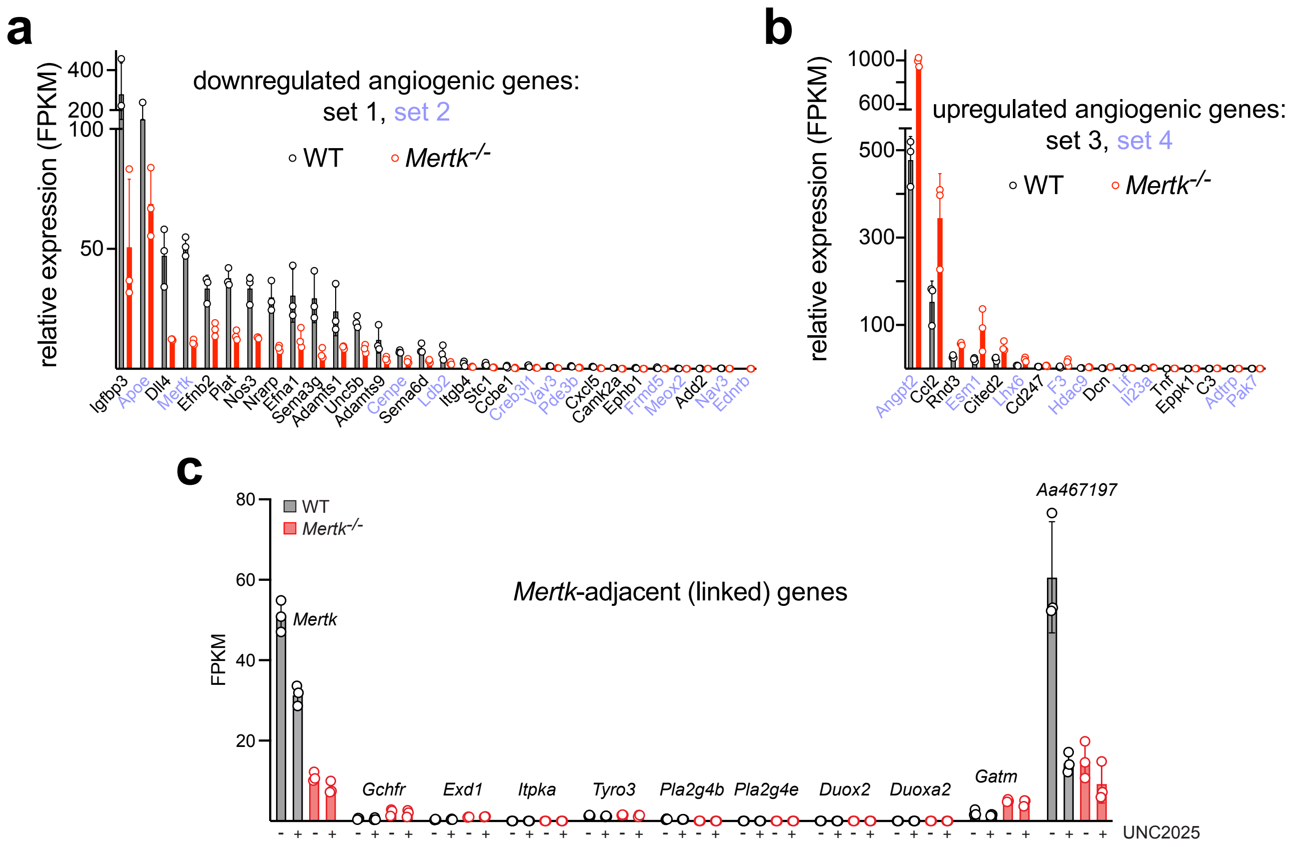

Supplementary Fig. 4. Relative expression levels of angiogenesis-related mRNAs in mouse BECs. (**a**) Expression levels of set 1/2 angiogenic genes significantly downregulated in *Mertk*$^{-/-}$ versus WT BECs analyzed by bulk RNAseq (see text). (**b**) Expression levels of set 3/4 angiogenic genes significantly upregulated in *Mertk*$^{-/-}$ versus WT BECs. (**c**) Expression levels of genes closely linked to *Mertk* in the mouse genome, in WT and *Mertk*$^{-/-}$ mouse BECs treated and untreated with UNC2025 (300 nM, 20 h). Each data point represents a separate BEC culture prepared from 8-10 mice.

Supplementary Fig. 5. Mer regulation of the mouse BEC proteome. (**a**) Heat maps of total mouse BEC proteome determined by mass spectrometry analyses of 3 culture replicates of WT versus *Mertk*$^{-/-}$ cells. (**b**) Panther GO pathway enrichment analysis of proteins downregulated in *Mertk*$^{-/-}$ BECs.

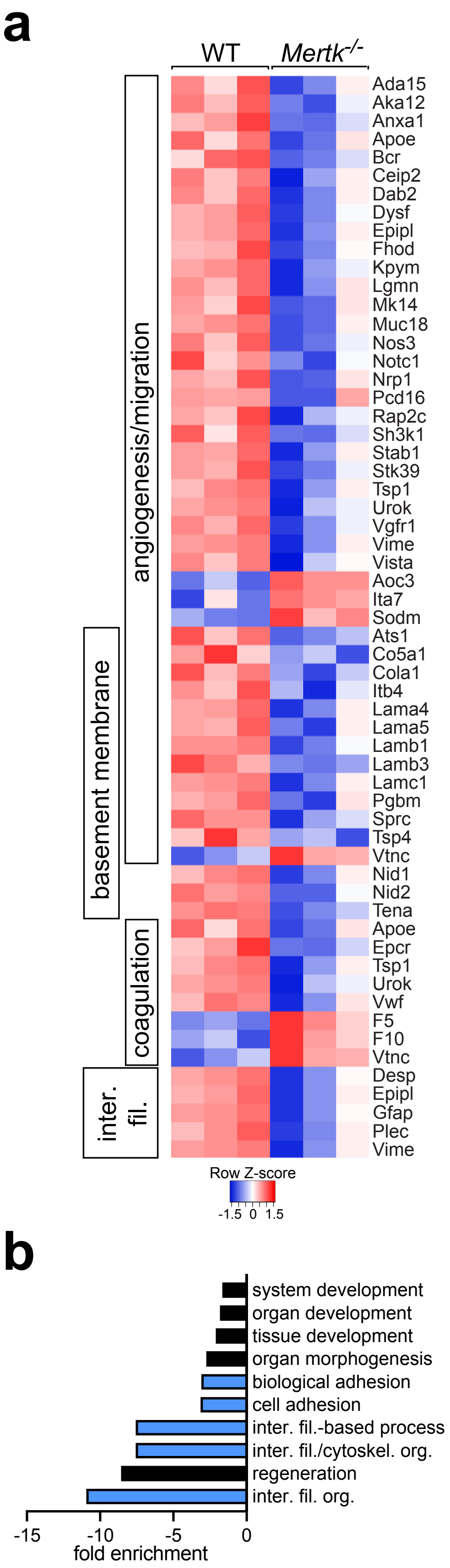

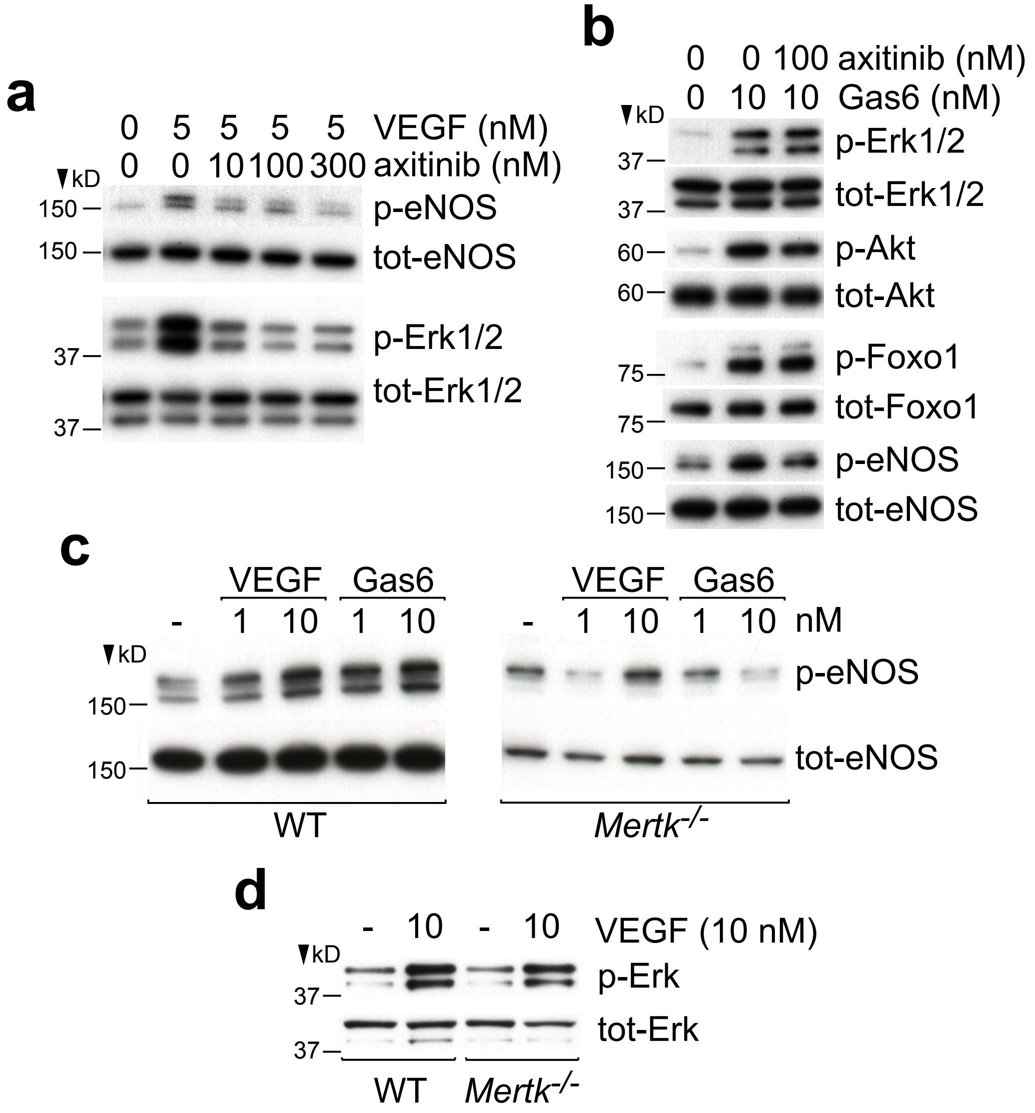

Supplementary Fig. 6. Mer and VEGFR signaling in BECs. (**a**) Inhibition of VEGF (5 nM, 15 min) stimulation of eNos and Erk1/2 phosphorylation upon 30 min pre-incubation with the indicated concentration of Axitinib, which inhibits all VEGFRs. (**b**) 30 min pre-incubation with Axitinib does not block Gas6 (10 nM, 15 min) stimulation of Erk1/2, Akt, Foxo1, or eNOS phosphorylation. (**c**) While Gas6 stimulation of eNOS phosphorylation is lost in *Mertk*$^{-/-}$ BECs, VEGF stimulation of eNOS phosphorylation is maintained. (**d**) VEGF stimulation of Erk1/2 phosphorylation is maintained in *Mertk*$^{-/-}$ BECs. All blots are

representative examples of experiments performed in three independent BEC cultures. (Quantification of multiple western blot runs in Supplementary Fig. 9.)

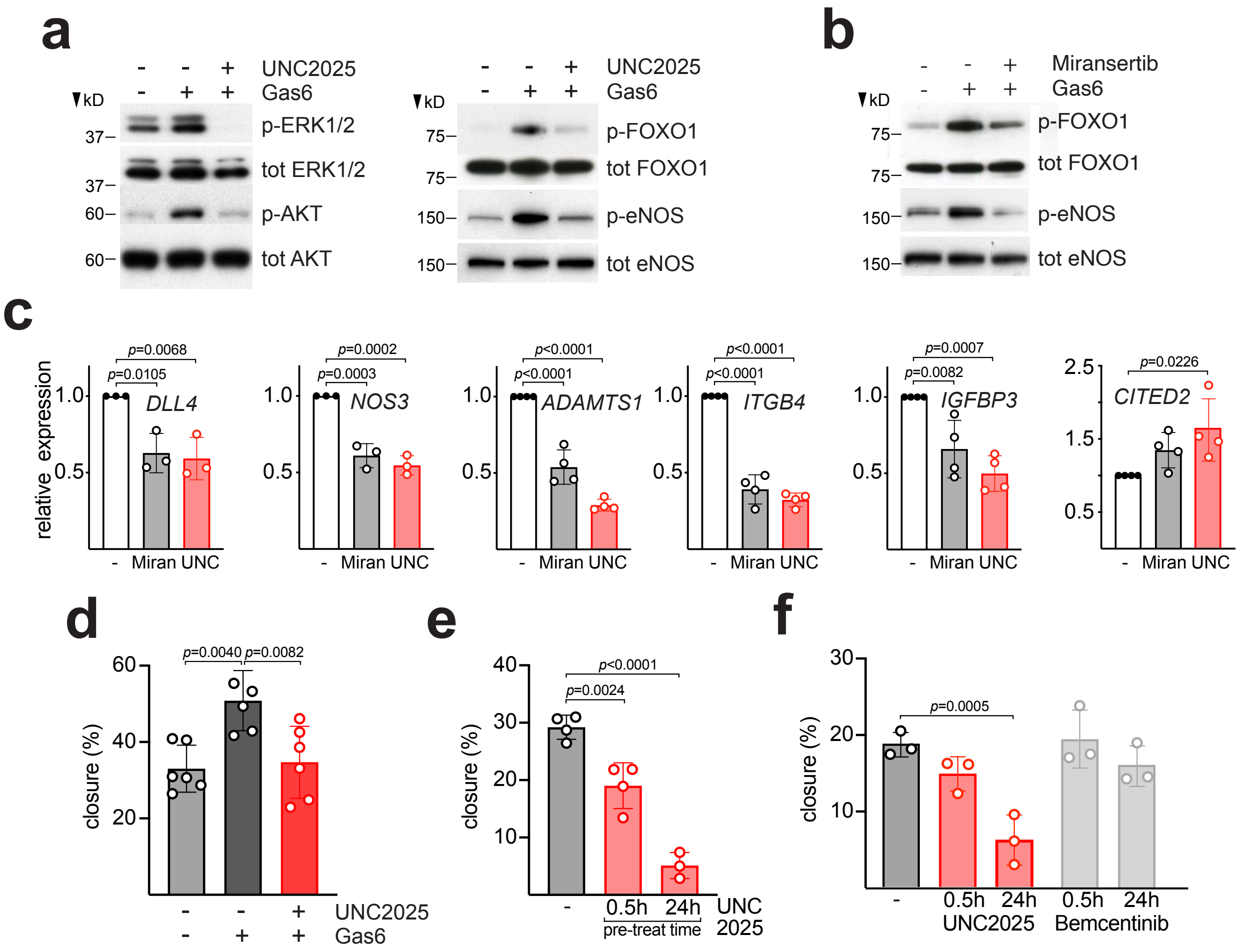

Supplementary Fig. 7. Mer regulation of human BEC physiology. (**a**) Representative western blots illustrating that Gas6 stimulation (10 nM, 15 min) of the phosphorylation of ERK1/2, AKT, FOXO1, and eNOS in human BECs is antagonized by 30 min prior treatment with the Mer inhibitor UNC2025 (300 nM), as it is in mouse BECs. (**b**) Representative western blot illustrating that Gas6 stimulation (10 nM, 15 min) of the phosphorylation of FOXO1 and eNOS in human BECs is antagonized by 30 min prior treatment with the Akt inhibitor Miransertib (100 nM), as it is in mouse BECs. (**c**) Expression of the indicated mRNAs in human BECs treated with Miransertib (100 nM,

20 h) or UNC2025 (300 nM, 20 h) relative to untreated cells, as determined by qRT-PCR (see Methods). (**d**) Percent closure of monolayers 5 h after the introduction of a scratch in human BECs (see Methods) - in untreated cells, cells treated with Gas6 (10 nM), or cells treated with Gas6 and UNC2025 (300 nM, added 30 min prior to Gas6). (**e**) Percent closure of monolayers 5 h after scratch in human BECs - in untreated cells, and cells pre-treated with UNC2025 (300 nM) for 0.5 and 24 h. (**f**) Percent closure of monolayers 5 h after the introduction of a scratch in human BECs - in untreated cells, and cells pre-treated with UNC2025 (300 nM) or Bemcentinib (300 nM) for 0.5 and 24 h. Graphs show mean +/- 1 SD, and data points indicate independently performed experiments. Statistical significance of differences was evaluated with a one-way ANOVA using a Dunnett's post test (c, d, e, f). (Quantification of multiple western blot runs in Supplementary Fig. 9.)

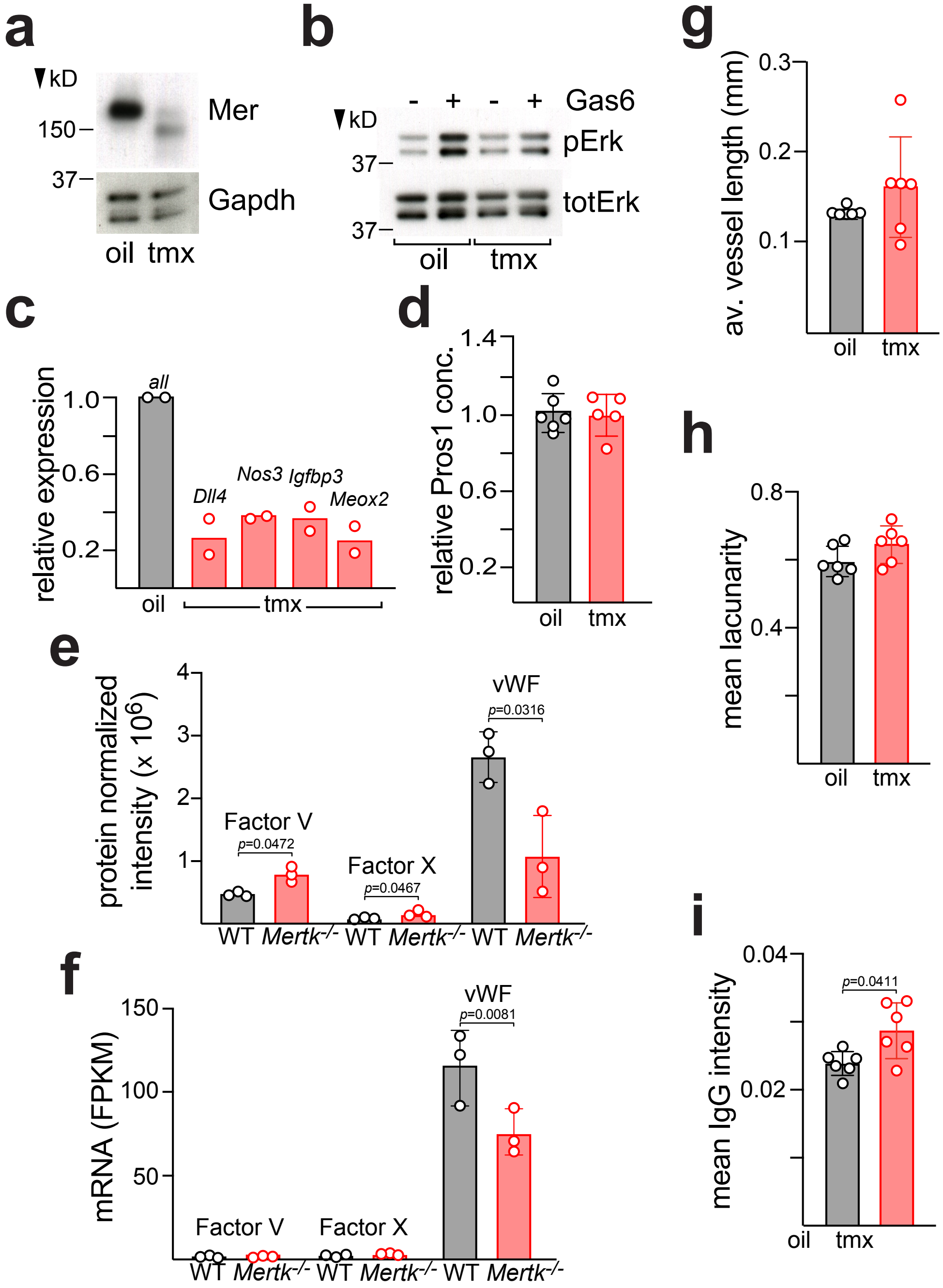

Supplementary Fig. 8. *In vivo* effects of EC-specific deletion of *Mertk*. (**a**) Representative western blot of BEC extracts prepared from corn oil (left) and tamoxifen (right, tmx)-injected *Cdh5Cre^{ER}Mertk$^{f/f}$* mice, 2 mo after injection, probed for Mer (top) and Gapdh (bottom) as a loading control. (**b**) Representative western blot of mouse BECs prepared from corn oil and tamoxifen (tmx)-injected *Cdh5Cre^{ER}Mertk$^{f/f}$* mice, 2 mo

after injection, incubated -/+ Gas6 (10 nM, 15 min), and probed for phospho-Erk1/2 (top) and (bottom) as a loading control. (**c**) Normalized expression of the indicated mRNAs in BEC cultures prepared from two independent sets of tamoxifen- versus corn oil-injected *Cdh5Cre^{ER}Mertk$^{f/f}$* mice, as measured by qRT-PCR. Confirmation of similar regulation of these mRNAs seen in *Mertk$^{-/-}$* versus WT BECs. (**d**) ELISA measurements of relative Pros1 levels (see Methods) in plasma isolated from corn oil (6 individuals) and tamoxifen (tmx)-injected (5 individuals) *Cdh5Cre^{ER}Mertk$^{f/f}$* mice. (**e**) Normalized protein levels of the clotting factors FV, FX, and vWF in extracts prepared from 3 separate cultures of WT and *Mertk$^{-/-}$* mouse BECs, as determined by MS (see Methods). (**f**) Levels of mRNAs encoding the clotting factors FV, FX, and vWF from 3 separate cultures of WT and *Mertk$^{-/-}$* mouse BECs, as determined by RNA-seq (see Methods). (**g**) Average vessel length and (**h**) mean lacunarity, as measured using AngioTool, in the lesion areas of corn oil and tamoxifen (tmx)-injected *Cdh5Cre^{ER}Mertk$^{f/f}$* mice, 14 d after the induction of photothrombotic stroke. (**i**) Mean immunoglobulin G (IgG) in the IBZ of corn oil and tamoxifen (tmx)-injected *Cdh5Cre^{ER}Mertk$^{f/f}$* mice, 14 d after the induction of photothrombotic stroke. Data points represent mean values of individual mice (see methods). In all graphs, data show mean +/- 1 SD. Statistical significance was evaluated using a Mann-Whitney test (i).

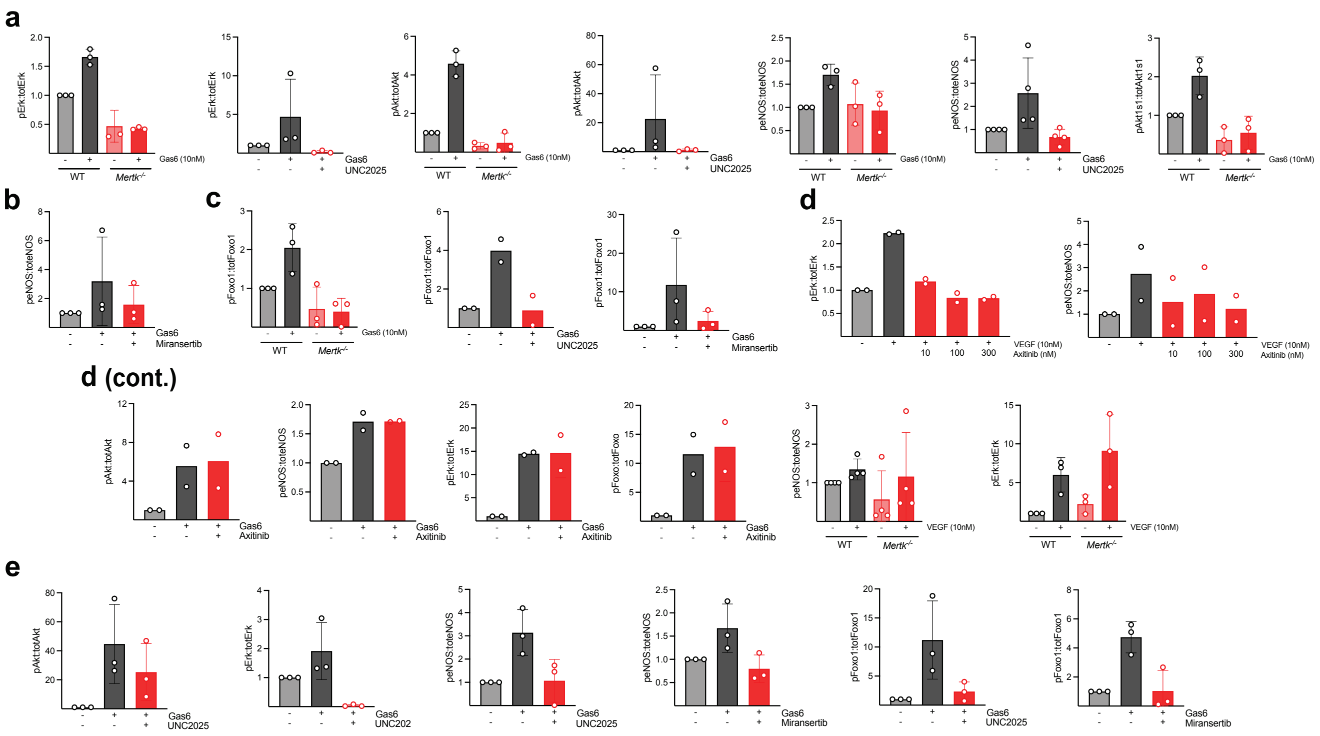

Supplementary Fig. 9. Western blot quantifications. (**a**) Quantification for representative blots presented in Fig. 1. Values normalized to no treatment (WT) in this and all subsequent panels. Each data point in this and all subsequent panels represents a separate western blot performed on separate batches of isolated BECs. (**b**) Blot of Supplementary Fig. 2. (**c**) Blots of Fig. 3. (**d**) Blots of Supplementary Fig. 6. (**e**) Blots of Supplementary Fig. 7.

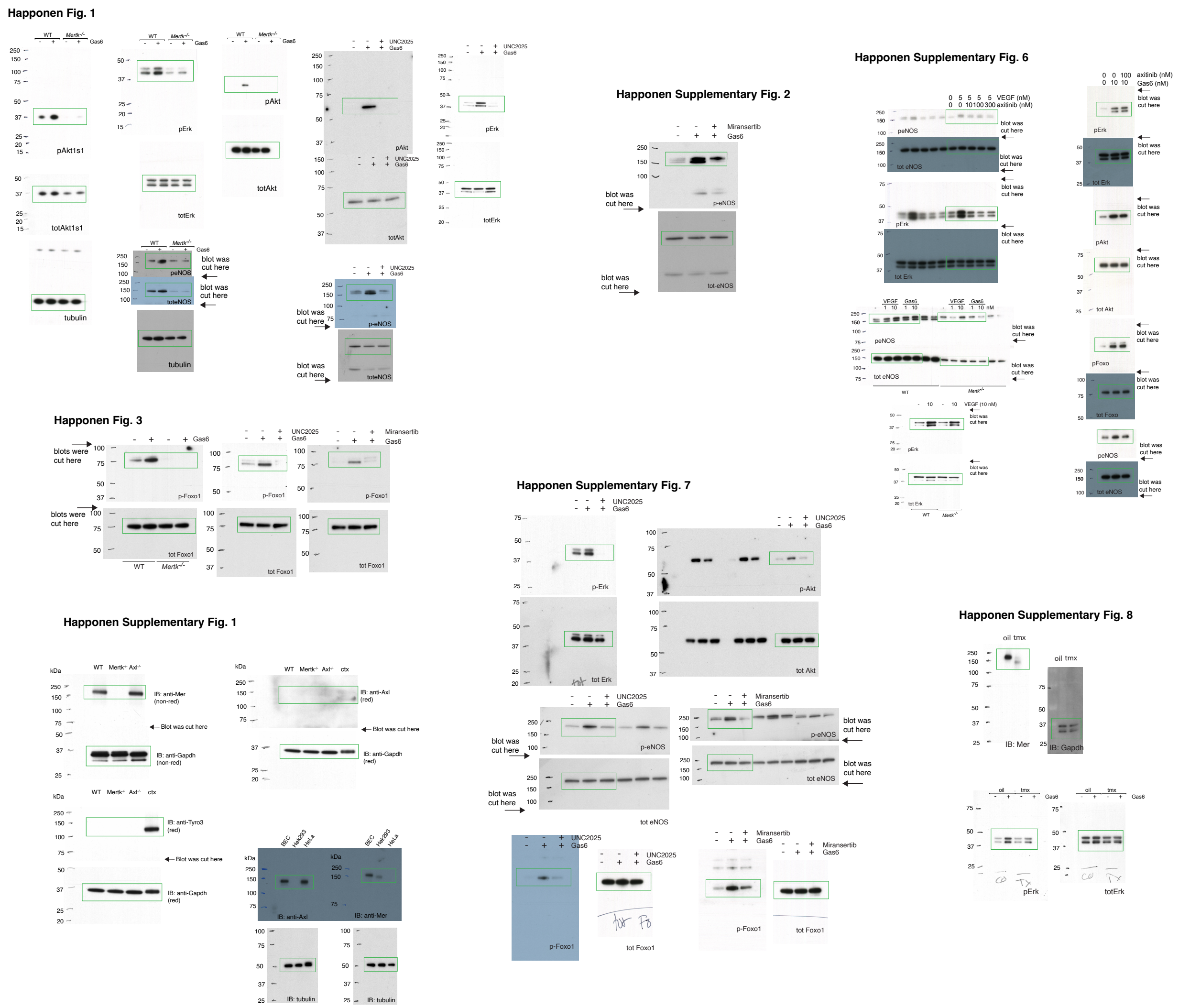

Supplementary Fig. 10. Unedited and uncropped Western blots for all figures and supplementary figures. Blots for Figs. 1 and 3, and Supplementary Figs. 1, 2, 6, 7, and 8, as indicated.
