# Supplementary material for: Regulation of brain endothelial cell physiology by the TAM receptor tyrosine kinase Mer
Source: Commun Biol. 2023 Sep 7;6:916. doi: 10.1038/s42003-023-05287-y (PMC10482977; doi:10.1038/s42003-023-05287-y)
Supplement: Supplementary file 2 — Description of Additional Supplementary Files [file 42003_2023_5287_MOESM2_ESM.docx]

**Description of Additional Supplementary Files**

**File name:** Supplementary Data 1

**Description:** This file contains source data for all graphs in the paper.
