# Supplementary material for: Regulation of brain endothelial cell physiology by the TAM receptor tyrosine kinase Mer
Source: Commun Biol. 2023 Sep 7;6:916. doi: 10.1038/s42003-023-05287-y (PMC10482977; doi:10.1038/s42003-023-05287-y)
Supplement: Supplementary file 4 — Reporting Summary [file 42003_2023_5287_MOESM4_ESM.pdf]

nature portfolio

Corresponding author(s): Greg Lemke

Last updated by author(s): 8/18./2023

Reporting Summary

Nature Portfolio wishes to improve the reproducibility of the work that we publish. This form provides structure for consistency and transparency in reporting. For further information on Nature Portfolio policies, see our Editorial Policies and the Editorial Policy Checklist.

Statistics

For all statistical analyses, confirm that the following items are present in the figure legend, table legend, main text, or Methods section.

n/a	Confirmed	
☐	☒	The exact sample size (*n*) for each experimental group/condition, given as a discrete number and unit of measurement
☐	☒	A statement on whether measurements were taken from distinct samples or whether the same sample was measured repeatedly
☐	☒	The statistical test(s) used AND whether they are one- or two-sided<br>*Only common tests should be described solely by name; describe more complex techniques in the Methods section.*
☐	☒	A description of all covariates tested
☐	☒	A description of any assumptions or corrections, such as tests of normality and adjustment for multiple comparisons
☐	☒	A full description of the statistical parameters including central tendency (e.g. means) or other basic estimates (e.g. regression coefficient) AND variation (e.g. standard deviation) or associated estimates of uncertainty (e.g. confidence intervals)
☐	☒	For null hypothesis testing, the test statistic (e.g. *F*, *t*, *r*) with confidence intervals, effect sizes, degrees of freedom and *P* value noted<br>*Give P values as exact values whenever suitable.*
☒	☐	For Bayesian analysis, information on the choice of priors and Markov chain Monte Carlo settings
☒	☐	For hierarchical and complex designs, identification of the appropriate level for tests and full reporting of outcomes
☒	☐	Estimates of effect sizes (e.g. Cohen's *d*, Pearson's *r*), indicating how they were calculated

Our web collection on statistics for biologists contains articles on many of the points above.

Software and code

Policy information about availability of computer code

Data collection	Confocal images were acquired with the Zen Blue software (version 2.5). Slide scanner images were acquired using Olympus VS-120 Virtual Slide Scanner and VS Desktop software (version 2.8). Transwell migration images were aquired with the Olympus BX40 microscope using the ProgRes CapturePro 2.10.0.1 software. QuantStudio Q5 was used for collecting qPCR data. ELISA plates and BCA assays were measured in the Tecan Infinite 200 PRO plate reader with the i-control software. For bulk RNAseq, the libraries were sequenced on the HiSeq 2500 system (Illumina) at single read 50bp. Image analysis and base calling were done with Illumina CASAVA-1.8.2. on HiSeq 2500 system and sequenced reads were quality-tested using FASTQC. The obtained reads were mapped to the mm10 genome using STAR v2.5.3a. Gene expression quantified with HOMER v 4.10.462 using the fragments per kilobase per million mapped reads (FPKM) normalization across exons of the top isoform. For phosphoproteomic analysis, the TMT labeled samples were analyzed on a Fusion Lumos mass spectrometer (Thermo), whereas total proteomic analysis was performed on an Orbitrap Eclipse mass spectrometer. Protein and peptide identification were done with Integrated Proteomics Pipeline – IP2. Tandem mass spectra were extracted from raw files using RawConverter and searched with ProLuCID against Swiss-Prot mouse database. The search space included all fully-tryptic and half-tryptic peptide candidates. Phosphorylation was considered as a variable modification on STY Data was searched with 50 ppm precursor ion tolerance and 600 ppm fragment ion tolerance. Identified proteins were filtered to 10 ppm precursor ion tolerance using DTASelect, with a target-decoy database search strategy to control the false discovery rate to 1% at the protein level. Quantitative analysis of TMT was done with Census, filtering reporter ions with 20 ppm mass tolerance and 0.6 isobaric purity filter. For more details on data acquisition in specific experiments, please refer to the materials and methods section.
Data analysis	Data was assembled and analyzed in Microsoft Excel, version 16.68, and graphs were plotted and analyzed for statistical significance in GraphPad Prism, version 9.5. Confocal microscopy and slide scanner images, as well as western blots, were processed in Fiji, version 2.3.0. Vascular parameters in microscopy images were analyzed with the AngioTool software (A computational tool for quantitative analysis of vascular networks. Zudaire E, Gambardella L, Kurcz C, Vermeren S. PLoS One. 2011;6(11):e27385. Epub 2011 Nov 16.) Other features of stroke

lesions were analyzed with CellProfiler version 4.2.1. Tuber formation was analyzed with the Angiogenesis Analyzer plugin for Fiji. For more details on data analysis in specific experiments, please refer to the materials and methods section.

For manuscripts utilizing custom algorithms or software that are central to the research but not yet described in published literature, software must be made available to editors and reviewers. We strongly encourage code deposition in a community repository (e.g. GitHub). See the Nature Portfolio guidelines for submitting code & software for further information.

Data

Policy information about availability of data

All manuscripts must include a data availability statement. This statement should provide the following information, where applicable:

- Accession codes, unique identifiers, or web links for publicly available datasets
- A description of any restrictions on data availability
- For clinical datasets or third party data, please ensure that the statement adheres to our policy

RNAseq data has been submitted to the Gene Expression Omnibus with the accession code GSE225474. The data will be embargoed for 4 years, but is accessible for reviewers using a specific token during this time. Mass spectrometry data has been deposited into MassIVE with the dataset number MSV000091355. The data will remain private until publication of the manuscript, but will be made accessible to reviewers using a specific token. All other data supporting the findings of the paper are found in the main text and supporting information. Raw data is available from the authors upon request.

Human research participants

Policy information about studies involving human research participants and Sex and Gender in Research.

Reporting on sex and gender	No human participants were used in this study
Population characteristics	No human participants were used in this study
Recruitment	No human participants were used in this study
Ethics oversight	No human participants were used in this study

Note that full information on the approval of the study protocol must also be provided in the manuscript.

Field-specific reporting

Please select the one below that is the best fit for your research. If you are not sure, read the appropriate sections before making your selection.

☒ Life sciences ☐ Behavioural & social sciences ☐ Ecological, evolutionary & environmental sciences

For a reference copy of the document with all sections, see nature.com/documents/nr-reporting-summary-flat.pdf

Life sciences study design

All studies must disclose on these points even when the disclosure is negative.

Sample size	Experiments on primary cells were carried out on 3 separate occasions on cells from separate cell isolations , each isolation originating from a pool of 8-10 mice. Within each experiment, a technical replicate of 2 was used. Cell stimulation and RNA harvest for Bulk RNAseq analysis was carried out at 3 separate occasions on cells from 3 separate cell isolations. Total proteomic analysis was done on primary cells from 3 separate isolations. Phosphoproteomic analysis by mass spectrometry was done on one technical replicate from one cell isolation, and validation of results by western blot was carried out on 3 separate occasions from cells originating from 3 separate isolations, where phosphospecific antibodies were available. Experiments on mice were carried out on at least 5-6 mice per group, as indicated in the figure legends. The number of mice in each group were determined based on previous publications using a similar stroke model (Labat-gest, V., Tomasi, S. Photothrombotic Ischemia: A Minimally Invasive and Reproducible Photochemical Cortical Lesion Model for Mouse Stroke Studies. J. Vis. Exp. (76), e50370, doi:10.3791/50370 (2013), Rust R., Weber R.Z., Grönnert L., Mulders G., Maurer M.A, Hofer A-S, Sartori A.M, Schwab M.E. Anti-Nogo-A antibodies prevent vascular leakage and act as pro-angiogenic factors following stroke. Sci Rep. 2019 Dec 27;9(1):20040, Weber RZ, Grönnert L, Mulders G, Maurer MA, Tackenberg C, Schwab ME and Rust R (2020) Characterization of the Blood Brain Barrier Disruption in the Photothrombotic Stroke Model. Front. Physiol. 11:586226. doi: 10.3389/fphys.2020.586226)
Data exclusions	no data was excluded
Replication	All experiments on cells were performed at least 3 times on cultures prepared from at least three separate cell isolations, originating from 8-10 mice each.
Randomization	Age- and sex- matched mice were used in all experiments, and littermates were randomly allocated to different treatment groups
Blinding	Analysis of mouse tissue was done in a semi-blinded manner, by assigning each mouse a number at the start of the experiment. The samples were analyzed only by mouse number, and the treatments allocated to each number was revealed after all the analysis was done.

Reporting for specific materials, systems and methods

We require information from authors about some types of materials, experimental systems and methods used in many studies. Here, indicate whether each material, system or method listed is relevant to your study. If you are not sure if a list item applies to your research, read the appropriate section before selecting a response.

Materials & experimental systems

n/a	Involved in the study	
☐	☒	Antibodies
☐	☒	Eukaryotic cell lines
☒	☐	Palaeontology and archaeology
☐	☒	Animals and other organisms
☒	☐	Clinical data
☒	☐	Dual use research of concern

Methods

n/a	Involved in the study	
☒	☐	ChIP-seq
☒	☐	Flow cytometry
☒	☐	MRI-based neuroimaging

Antibodies

Antibodies used

Mouse antibodies:,
anti-Akt (pan), #4691 from Cell Signaling, dilution 1:1000 for western blot
anti-p-Akt (S473), #4058 from Cell Signaling, dilution 1:1000 for western blot
anti-Akt1s1, #2691 from Cell Signaling, dilution 1:1000 for western blot
anti-p-Akt1s1 (T246) #2997 from Cell Signaling, dilution 1:1000 for western blot
anti-Axl #AF854 fromR&D systems, dilution 1:1000 for western blot
anti-CD31, #AF3628 from R&D systems, dilution 1:200 for IHC
anti-eNOS #32027 from Cell Signaling, dilution 1:1000 for western blot
anti-p-eNOS (S1177) #9571 from Cell Signaling, dilution 1:1000 for western blot
anti-Foxo1 #2880 from Cell Signaling, dilution 1:1000 for western blot and 1:100 for ICC
anti-p-Foxo1 (S256) #84192 from Cell Signaling, dilution 1:1000 for western blot
anti-Iba1 #019-19741 from Wako, dilution 1:100 for IHC
anti-Map2 #ab5392 from Abcam, dilution 1:10 000 for IHC
anti-Mer #DS5MMR from eBiosciences, dilution 1:2000 for western blot and 1:200 for IHC
anti-Tyro3 #5585 from Cell Signaling, dilution 1:500 for western blot
anti-Protein S #MAB4976 from R&D systems, dilution 1:500 for ELISA
anti-P44/42 #4695 from Cell Signaling, dilution 1:1000 for western blot
anti-p-P44/42 (T202/Y204) #9101 from Cell Signaling, dilution 1:1000 for western blot

Human antibodies
anti-Akt (pan) #4691 from Cell Signaling, dilution 1:1000 for western blot
anti-p-Akt (S473) #4058 from Cell Signaling, dilution 1:1000 for western blot
anti-Axl #H-124 from Santa Cruz, dilution 1:1000 for western blot
anti-eNOS #32027 from Cell Signaling, dilution 1:1000 for western blot
anti-p-eNOS (S1177) #9570 from Cell Signaling, dilution 1:1000 for western blot
anti-Foxo1 #2880 from Cell Signaling, dilution, 1:1000 for western blot
anti p-Foxo1 (S256) #84192 from Cell Signaling, dilution 1:1000 for western blot
anti-Mer #ab52968 from Abcam, dilution 1:2000 for western blot
anti-Protein S #A0384 from Dako, dilution 1:420 for ELISA
anti-P44/42 #4695 from Cell Signaling, dilution 1:1000 for western blot
anti-p-p44/42 (T202/Y204) #9101 from Cell Signaling, dilution 1:1000 for western blot

Validation

Antibodies were considered validated based on the statements and data provided by the manufacturers, as well as by usage in this manuscript. Application key as follows: WB-Western Blot IP-Immunoprecipitation IHC-Immunohistochemistry ChIP-Chromatin Immunoprecipitation C&R-CUT&RUN C&T-CUT&Tag DB-Dot Blot eCLIP-eCLIP IF-Immunofluorescence F-Flow Cytometry

anti-Akt (pan) (#4691, Cell Signaling) validated for WB, IP, IHC, IF and F in Human, Mouse, Rat, Monkey and D. melanogaster, as described by the manufacturer https://www.cellsignal.com/products/primary-antibodies/akt-pan-c67e7-rabbit-mab/4691

anti-p-Akt (S473) (#4058, Cell Signaling) validated for WB, IP, IF and F in Human, Mouse and Rat, as described by the manufacturer https://www.cellsignal.com/products/primary-antibodies/phospho-akt-ser473-193h12-rabbit-mab/4058

anti-Akt1s1 (#2691, Cell Signaling) validated for WB, IP and IHC in Human, Mouse, Rat and Monkey, as described by the manufacturer https://www.cellsignal.com/products/primary-antibodies/pras40-d23c7-xp-rabbit-mab/2691

anti-p-Akt1s1 (T246) (#2997, Cell Signaling) validated for WB, IP and IHC in Human, Mouse, Rat and Monkey, as described by the manufacturer https://www.cellsignal.com/products/primary-antibodies/phospho-pras40-thr246-c77d7-rabbit-mab/2997

anti-Axl (#AF854, R&D systems), validated for ELISA , IHC and WB in Mouse, as described by the manufacturer. Further validation on knock out tissues performed in our laboratory and in this paper. https://www.rndsystems.com/products/mouse-axl-antibody_af854

anti-Axl (#H-124, Santa Cruz), raised against an epitope in human Axl. The product has been discontinued, but has shown reactivity to Axl in numerous publications (for instance Happonen K.E., Tran S., Mörgelin M., Prince R., Calzavarini S., Angelillo-Scherrer A., Dahlbäck B., The Gas6-Axl Protein Interaction Mediates Endothelial Uptake of Platelet Microparticles. J Biol Chem . 2016 May 13;291(20):10586-601 and Gustafsson A., Fritz H., Dahlbäck B., Gas6-Axl signaling in presence of Sunitinib is enhanced, diversified and sustained in renal tumor cells, resulting in tumor-progressive advantages. Exp Cell Res . 2017 Jun 1;355(1):47-56. doi: 10.1016/j.yexcr.2017.03.040.) https://www.scbt.com/p/axl-antibody-h-124

anti-CD31 (#AF3628, R&D systems), validated for ELISA, WB, IHC, ICC and F in Mouse, as described by the manufacturer. Verified endothelial staining in this paper. https://www.rndsystems.com/products/mouse-rat-cd31-pecam-1-antibody_af3628

anti-eNOS (#32027, Cell Signaling), validated for WB, IP, ICC in Human, Mouse, Rat and Bovine, as described by the manufacturer https://www.cellsignal.com/products/primary-antibodies/enos-d9a5l-rabbit-mab/32027

anti-p-eNOS (S1177) (#9571, Cell Signaling), validated for WB in Human, Bovine and Pig, as described by the manufacturer, predicted 100% reactivity with Mouse and Rat due to sequence similarity. Validated for Mouse eNOS in WB in this paper. https://www.cellsignal.com/products/primary-antibodies/phospho-enos-ser1177-antibody/9571

anti-Foxo1 (#2880, Cell Signaling), validated for WB, IP, IHC, ICC, F, ChIP in Human, Mouse, Rat and Monkey, as described by the manufacturer. https://www.cellsignal.com/products/primary-antibodies/foxo1-c29h4-rabbit-mab/2880

anti-p-Foxo1 (S256) (#84192, Cell Signaling), validated for WB and IP in Human, Mouse, Rat and Monkey, as described by the manufacturer. https://www.cellsignal.com/products/primary-antibodies/phospho-foxo1-ser256-e1f7t-rabbit-mab/84192

anti-Iba1 (#019-19741, Wako), validated for ICC and IHC in Human, Mouse and Rat as described by the manufacturer. Verified microglial staining in this paper as well as in Huang Y., Happonen K.E., Burrola P., O'Connor C., Hah N., Huang L., Nimmerjahn A., Lemke G., Microglia use TAM receptors to detect and engulf amyloid β plaques. Nat Immunol. 2021 May;22(5):586-594 https://labchem-wako.fujifilm.com/us/product/detail/W01W0101-1974.html

anti-Map2 (#ab5392, Abcam), validated for ICC and WB in Mouse and Rat, as described by the manufacturer. https://www.abcam.com/products/primary-antibodies/map2-antibody-ab5392.html

anti-Mer (#DS5MMR, eBiosciences), validated for WB, IHC, ICC, F and IP mouse, validation performed on knock out tissues, as described by the manufacturer. Further validation performed in our laboratory and in this paper. https://www.thermofisher.com/antibody/product/MERTK-Antibody-clone-DS5MMER-Monoclonal/14-5751-82

anti-Mer (#ab52968, Abcam), validated for WB, IP, IHC on Human, validation done on knock out cells. https://www.abcam.com/products/primary-antibodies/mertk-antibody-y323-ab52968.html

anti-Tyro3 (#5585, Cell Signaling), validated for WB and IP in Human, Mouse, Rat and Monkey, as described by the manufacturer. https://www.cellsignal.com/products/primary-antibodies/tyro3-d38c6-rabbit-mab/5585

anti-Protein S (#MAB4976, R&D systems), validated for ELISA and WB in Mouse, as described by the manufacturer. https://www.rndsystems.com/products/mouse-protein-s-pros1-antibody-818002_mab4976

anti-Protein S (#A0384, Dako), no validation provided on manufacturer's website, but validated for the use in ELISA in this paper as well as in Calzavarini S., Prince-Eladnani R., Saller F., Bologna L., Burnier L., Brisset A.C., Quarroz C., Reina Caro M.D., Ermolayev V., Matsumura Y., Fernandez J.A., Hackeng T.M., Griffin J.H., Angelillo-Scherrer A. Platelet protein S limits venous but not arterial thrombosis propensity by controlling coagulation in the thrombusBlood. 2020 May 28; 135(22): 1969–1982 and for flow cytometry in Happonen K.E., Tran S., Mörgelin M., Prince R., Calzavarini S., Angelillo-Scherrer A., Dahlbäck B., The Gas6-Axl Protein Interaction Mediates Endothelial Uptake of Platelet Microparticles. J Biol Chem. 2016 May 13;291(20):10586-601

anti-P44/42 (#4695, Cell Signaling), validated for WB, IP, IHC, IF and F, in Human, Mouse, Rat, Hamster, Monkey, Mink, D. melanogaster, Zebrafish, Bovine, Dog, Pig and C. elegans, as described by the manufacturer. https://www.cellsignal.com/products/primary-antibodies/p44-42-mapk-erk1-2-137f5-rabbit-mab/4695

anti-p-P44/42 (T202/Y204) (#9101, Cell Signaling), validated for WB, IP, IF and F in Human, Mouse, Rat, Hamster, Monkey, Mink, D. melanogaster, Zebrafish, Bovine, Pig, C. elegans, as described by the manufacturer. https://www.cellsignal.com/products/primary-antibodies/phospho-p44-42-mapk-erk1-2-thr202-tyr204-antibody/9101

Eukaryotic cell lines

Policy information about cell lines and Sex and Gender in Research

Cell line source(s)	Primary Human Brain Microvascular Endothelial Cells (ACBRI 376) were from Cell Systems (https://cell-systems.com/products/human-brain-microvascular-endothelial-cells-acbri-376?variant=37945739019)
Authentication	The cells were not authenticated
Mycoplasma contamination	The cells were not tested for mycoplasma contamination
Commonly misidentified lines (See ICLAC register)	*Name any commonly misidentified cell lines used in the study and provide a rationale for their use.*

Animals and other research organisms

Policy information about studies involving animals; ARRIVE guidelines recommended for reporting animal research, and Sex and Gender in Research

Laboratory animals	All mice were on a pure C57Bl/6 background. The Mertk-/- and Axl-/- strains (Q. Lu et al., Tyro-3 family receptors are essential regulators of mammalian spermatogenesis. Nature 398, 723-728 (1999)), Mertkf/f strain (L. Fourgeaud et al., TAM receptors regulate multiple features of microglial physiology. Nature 532, 240-244 (2016)) and Cdh5CreER strain (Y. Wang et al., Ephrin-B2 controls VEGF-induced angiogenesis and lymphangiogenesis. Nature 465, 483-486 (2010)) have been described previously. Mice with an endothelial specific knock out of Mertk were generated by crossing the Mertkf/f and Cdh5CreER strains. All experiments were carried out on 2-4 month old mice.
Wild animals	The study did not involve wild animals
Reporting on sex	Both male and female mice were used in the experiments, allocating sex- and age matched mice to each group. No evident difference was found when analyzing sexes separately
Field-collected samples	No field-collected samples were used in this study
Ethics oversight	All experiments and procedures were conducted according to the guidelines established by the Institutional Animal Care and Use Committee (IACUC) (protocol nr. 17-00046)

Note that full information on the approval of the study protocol must also be provided in the manuscript.
